# Family History of Early Infant Death Correlates with Earlier Age at Diagnosis But Not Shorter Time to Diagnosis for Severe Combined Immunodeficiency

**DOI:** 10.3389/fimmu.2017.00808

**Published:** 2017-07-12

**Authors:** Anderson Dik Wai Luk, Pamela P. Lee, Huawei Mao, Koon-Wing Chan, Xiang Yuan Chen, Tong-Xin Chen, Jian Xin He, Nadia Kechout, Deepti Suri, Yin Bo Tao, Yong Bin Xu, Li Ping Jiang, Woei Kang Liew, Orathai Jirapongsananuruk, Tassalapa Daengsuwan, Anju Gupta, Surjit Singh, Amit Rawat, Amir Hamzah Abdul Latiff, Anselm Chi Wai Lee, Lynette P. Shek, Thi Van Anh Nguyen, Tek Jee Chin, Yin Hsiu Chien, Zarina Abdul Latiff, Thi Minh Huong Le, Nguyen Ngoc Quynh Le, Bee Wah Lee, Qiang Li, Dinesh Raj, Mohamed-Ridha Barbouche, Meow-Keong Thong, Maria Carmen D. Ang, Xiao Chuan Wang, Chen Guang Xu, Hai Guo Yu, Hsin-Hui Yu, Tsz Leung Lee, Felix Yat Sun Yau, Wilfred Hing-Sang Wong, Wenwei Tu, Wangling Yang, Patrick Chun Yin Chong, Marco Hok Kung Ho, Yu Lung Lau

**Affiliations:** ^1^LKS Faculty of Medicine, Department of Paediatrics and Adolescent Medicine, The University of Hong Kong, Hong Kong, Hong Kong; ^2^Shenzhen Primary Immunodeficiency Diagnostic and Therapeutic Laboratory, The University of Hong Kong-Shenzhen Hospital, Shenzhen, China; ^3^Guangzhou Children’s Hospital, Guangzhou, China; ^4^Department of Allergy and Immunology, Shanghai Children’s Medical Center, Shanghai Jiao Tong University School of Medicine, Shanghai, China; ^5^Beijing Children’s Hospital, Capital Medical University, Beijing, China; ^6^Institut Pasteur d’Algérie, Algeria, North Africa; ^7^Allergy Immunology Unit, Advanced Pediatrics Centre, Postgraduate Institute of Medical Education and Research, Chandigarh, India; ^8^Guang Zhou Women and Children’s Medical Center, Guangzhou, China; ^9^Children’s Hospital of Chongqing Medical University, Chongqing, China; ^10^KK Women’s and Children’s Hospital, Singapore, Singapore; ^11^Department of Pediatrics, Siriraj Hospital Mahidol University, Bangkok, Thailand; ^12^Queen Sirikit National Institute of Child Health, Bangkok, Thailand; ^13^Monash University, Selangor, Malaysia; ^14^Mount Elizabeth Hospital, Singapore, Singapore; ^15^National University of Singapore, Singapore, Singapore; ^16^National Children’s Hospital, Hanoi, Vietnam; ^17^Sarawak General Hospital Malaysia, Kuching, Malaysia; ^18^National Taiwan University Children’s Hospital, Taipei, Taiwan; ^19^University Kebangsaan Malaysia Medical Center, Kuala Lumpur, Malaysia; ^20^Sichuan Second West China Hospital, Sichuan, China; ^21^Department of Paediatrics, Holy Family Hospital, New Delhi, India; ^22^Department of Immunology, Institut Pasteur de Tunis and University Tunis-El Manar, Tunis, Tunisia; ^23^Faculty of Medicine, Department of Paediatrics, University of Malaya, Kuala Lumpur, Malaysia; ^24^San Pedro Hospital, Davao, Philippines; ^25^Children’s Hospital of Fudan University, Shanghai, China; ^26^The First Affiliated Hospital, Sun Yat-sen University, Guangzhou, China; ^27^Nanjing Children’s Hospital, Nanjing, China; ^28^Queen Elizabeth Hospital, Hong Kong, Hong Kong

**Keywords:** severe combined immunodeficiency, family history, candidiasis, absolute lymphocyte count, newborn screening

## Abstract

**Background:**

Severe combined immunodeficiency (SCID) is fatal unless treated with hematopoietic stem cell transplant. Delay in diagnosis is common without newborn screening. Family history of infant death due to infection or known SCID (FH) has been associated with earlier diagnosis.

**Objective:**

The aim of this study was to identify the clinical features that affect age at diagnosis (AD) and time to the diagnosis of SCID.

**Methods:**

From 2005 to 2016, 147 SCID patients were referred to the Asian Primary Immunodeficiency Network. Patients with genetic diagnosis, age at presentation (AP), and AD were selected for study.

**Results:**

A total of 88 different SCID gene mutations were identified in 94 patients, including 49 *IL2RG* mutations, 12 *RAG1* mutations, 8 *RAG2* mutations, 7 *JAK3* mutations, 4 *DCLRE1C* mutations, 4 *IL7R* mutations, 2 *RFXANK* mutations, and 2 *ADA* mutations. A total of 29 mutations were previously unreported. Eighty-three of the 94 patients fulfilled the selection criteria. Their median AD was 4 months, and the time to diagnosis was 2 months. The commonest SCID was X-linked (*n* = 57). A total of 29 patients had a positive FH. Candidiasis (*n* = 27) and bacillus Calmette–Guérin (BCG) vaccine infection (*n* = 19) were the commonest infections. The median age for candidiasis and BCG infection documented were 3 months and 4 months, respectively. The median absolute lymphocyte count (ALC) was 1.05 × 10^9^/L with over 88% patients below 3 × 10^9^/L. Positive FH was associated with earlier AP by 1 month (*p* = 0.002) and diagnosis by 2 months (*p* = 0.008), but not shorter time to diagnosis (*p* = 0.494). Candidiasis was associated with later AD by 2 months (*p* = 0.008) and longer time to diagnosis by 0.55 months (*p* = 0.003). BCG infections were not associated with age or time to diagnosis.

**Conclusion:**

FH was useful to aid earlier diagnosis but was overlooked by clinicians and not by parents. Similarly, typical clinical features of SCID were not recognized by clinicians to shorten the time to diagnosis. We suggest that lymphocyte subset should be performed for any infant with one or more of the following four clinical features: FH, candidiasis, BCG infections, and ALC below 3 × 10^9^/L.

## Introduction

Severe combined immunodeficiency (SCID) is a group of genetic diseases causing profound developmental and functional impairment of T cells, affecting cellular and humoral immunities. Currently, at least 49 genes are identified to be responsible for SCID and its variants (1–3). Of all the SCID genes, the commonest gene involved is the IL-2 receptor gamma chain gene (*IL2RG*), which accounted for 45 and 19% of SCID cases before and after the T-cell receptor excision circle (TREC) newborn screening, and was introduced in USA (3–5). Patients typically present with recurrent infections from opportunistic pathogens and live-attenuated vaccines, such as bacillus Calmette–Guérin (BCG) (6), chronic diarrhea, and failure to thrive (FTT), eventually die within the first 2 years of life if left untreated (7). Patients typically have low absolute lymphocyte count (ALC). They have been classified by the number of B lymphocytes as B+ or B− and recently by the causative genetic mutation.

The definitive treatment for SCID is hematopoietic stem cell transplant (HSCT). In addition, gene therapy serves as an alternative for X-linked and adenosine deaminase (ADA)-deficient SCID if suitable HSC donors are not available (8). SCID patients have a 94% survival rate if they undergo HSCT within the first 3.5 months of life (9). To facilitate timely HSCT, an early diagnosis must be made. However, delay in diagnosis is common due to the lack of awareness of the distinctive presenting features of SCID, such as recurrent and persistent opportunistic infections (2). To date, the only feature that is associated with an earlier diagnosis is a positive family history of infant death due to infection or known SCID in USA (10). In addition, family history of SCID is associated with earlier HSCT before 3.5 months (9, 11). Our present study aimed to identify the clinical features that could help clinicians diagnose SCID earlier by comparing the age and time to the diagnosis of patients with or without certain clinical features.

## Materials and Methods

### Patient Source and Selection

The Asian Primary Immunodeficiency Network (APIN) is a primary immunodeficiency (PID) referral network established in 2009 by The University of Hong Kong as a platform for consultation and offering free genetic testing for suspected PID in over 70 centers in Asia and Africa. Its database stores clinical information provided by the referring doctors, laboratory results, and genetic test reports (12–14). From 2005 to 2016, 147 SCID patients were referred from 23 centers to the APIN for consultation and genetic testing, 42 of whom were reported in our previous study (13). In our study, we included patients with documented age at presentation (AP) and diagnosis. Among them, we selected patients with genetic diagnosis for identifying factors that affected age and time to diagnosis of SCID.

### Data Collection

**Figure 1:**
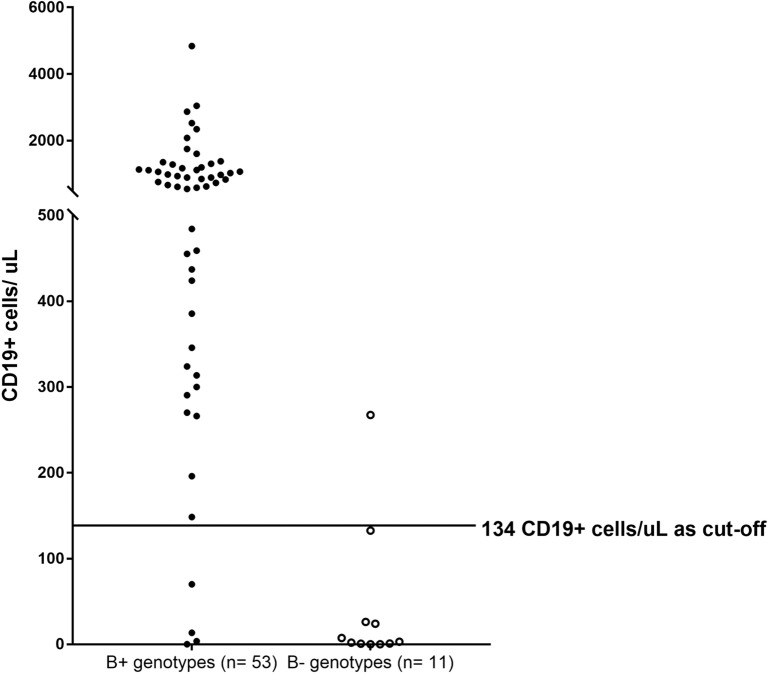
CD19^+^ cell counts of patients with B+ and B− genotypes. B+ genotypes group consisted of CD19^+^ cell counts of patients with mutations found in *IL2RG* (*n* = 43), *IL7R* (*n* = 3), and *JAK3* (*n* = 5). B− genotypes group consisted of CD19^+^ cell counts of patients with mutations found in *ADA* (*n* = 1), *DCLRE1C* (*n* = 3), *RAG1* (*n* = 3), and *RAG2* (*n* = 2). The cutoff for distinguishing B+ and B− patients was 134 CD19^+^ cells/μL. Three patients with *IL2RG* mutations were classified as having B− SCID.

The referring doctors provided the clinical records of patients, together with the laboratory results. Demographic data, clinical presentation, and progress as well as investigation results including ALC and lymphocyte subsets of the patients were recorded. We only considered the clinical features and progression before the diagnosis of SCID was made. We defined AP as the age when the first clinical symptom was documented in the clinical record, age at diagnosis (AD) as the age when clinical diagnosis of SCID was made, and time to diagnosis as the duration between AP and AD. We only considered patients to have certain clinical feature if that feature was stated in the referral summary. We defined recurrent infections as more than one episode of infections affecting similar systems. We considered the infection to be severe if at least one of the following was present: life-threatening complications (such as acute respiratory distress syndrome and sepsis), intensive care unit (ICU) admission, and life support being used (intubation, ventilation, and resuscitation). We defined opportunistic infection as an infection with at least one of the following pathogens was involved: BCG, *Candida, Pseudomonas aeruginosa, Acinetobacter baumannii, Pneumocystis jiroveci* (PCP), *Aspergillus*, cytomegalovirus (CMV), and herpes zoster virus. We defined opportunistic infections by *Candida* if patients were documented to have invasive candidiasis, candidemia or persistent oral candidiasis (15). We presented the ALC recorded at the time of SCID diagnosis. Patients were said to have lymphopenia if ALC below 3 × 10^9^/L as described previously (10). Since there is no universally agreed cutoff of B-cell number to distinguish B+ and B− SCID, we defined the cut-off as 134/μL based on the CD19^+^ B-cell counts of patients with B+ genotypes (*IL2RG, IL7R*, and *JAK3*) and those with B− genotypes (*ADA, DCLRE1C, RAG1*, and *RAG2*) (Figure 1).

Genetic analysis was performed in the Department of Pediatrics and Adolescent Medicine of the University of Hong Kong using PCR and direct sequencing (Table SE1 in Supplementary Material) (13). Genetic and functional studies on PID, data archival in the APIN database, and DNA storage were approved by the Clinical Research Ethics Review Board of the University of Hong Kong and Queen Mary Hospital (Ref. no. UW 08-301) in accordance with the Declaration of Helsinki, with written informed consent obtained from parents of subjects. HGMD Pro version 2016.4 (16) and Immunodeficiency mutation databases (IDbases) (17) were used to identify unreported mutations. The nomenclatures of cDNA mutations were based on coding region. For each unreported mutation, the population frequency was analyzed by Exome Aggregation Consortium Browser (18). Effects of missense mutations on protein functions were predicted by PANTHER (19), PHD-SNP (20), SIFT (21), SNAP (22), Meta-SNP (23), and PolyPhen2 (24). The protein structure predicted to be involved was identified using NCBI Protein database (25) and UniProt Knowledgebase database (26).

### Statistical Analysis

For descriptive statistics, all data were expressed in median and range (month). Univariate analysis was performed using Mann–Whitney *U* test; multivariate linear regression was performed for all factors that were significant (*p* < 0.05) in univariate analysis. We defined statistical significance as *p* < 0.05, and 95% confidence interval did not contain 0 in multivariate analysis. We did not include opportunistic infection group in the multivariate linear regression to avoid multicollinearity.

Patients with missing categorical data such as clinical features were considered to be without the features. Patients with missing numerical data such as ALC were not analyzed when analyzing median and range.

## Results

### Patients Selection

**Figure 2:**
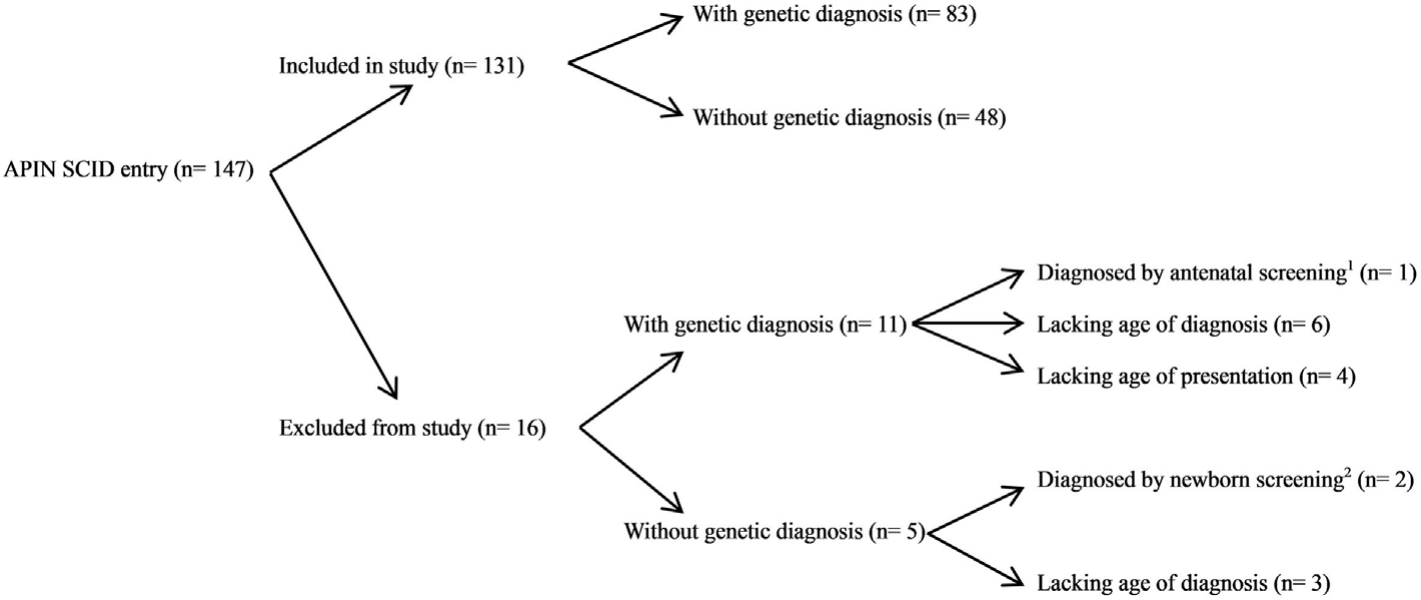
Patients selection algorithm in this study. From 147 SCID entries in the Asian Primary Immunodeficiency Network (APIN) database, 131 patients were included in our study and 16 patients were excluded from our study. Three patients were excluded as they were diagnosed by screening either antenatally or at birth. Thirteen patients were excluded due to the lack of age at presentation (*n* = 4) or the lack of age at diagnosis (*n* = 9). ^1^Cordocentesis was performed due to positive family history of SCID, revealed low CD4^+^ count. ^2^Complete blood count, lymphocyte subsets, and immunoglobulins measurement were performed in one patient due to positive family history, revealed severe T- and B-cell lymphopenia and low serum IgA and IgM; newborn T-cell receptor excision circle (TREC) screening revealed 0 TREC copy in another patient.

From 147 SCID patients referred to the APIN, 131 of them had documented AP and diagnosis. Among these patients, 83 of them had genetic diagnosis (Figure 2). Sixteen patients were excluded from the study due to the lack of AP (*n* = 4), the lack of AD (*n* = 9), and being diagnosed by screening (*n* = 3). Among the 16 patients excluded from the study, 11 of them had genetic diagnosis. Altogether, molecular diagnosis of SCID was identified in 94 patients in our cohort.

### Genetic Mutations in Patients

The genetic mutations of the 83 SCID patients included in our study and 11 SCID patients excluded from our study are shown in Table 1 and Table SE1 in Supplementary Material, respectively. The commonest gene identified was *IL2RG* (*n* = 65), followed by *RAG1* (*n* = 7), *RAG2* (*n* = 7), *JAK3* (*n* = 5), *DCLRE1C* (*n* = 4), *IL7R* (*n* = 3), *RFXANK* (*n* = 2), and *ADA* (*n* = 1). Eighty-eight different mutations were identified in this study (49 *IL2RG* mutations, 12 *RAG1* mutations, 8 *RAG2* mutations, 7 *JAK3* mutations, 4 *DCLRE1C* mutations, 4 *IL7R* mutations, 2 *RFXANK* mutations, and 2 *ADA* mutations). There was no difference in clinical features between X-linked and autosomal recessive SCID patients (Table SE2 in Supplementary Material).

**Table 1:** Genetic mutations of SCID patients (*n* = 83).

No	Gene	Intron (I)/exon (E)	Nucleotide change	Predicted change
P001	*IL2RG*	E1	c.3G>T	M1I
P002	*IL2RG*	E2	c.127delA	T43fsX70
P003	*IL2RG*	E2	c.202G>T	E68X
P004a	*IL2RG*	E2	c.202G>A	E68K
P004b	*IL2RG*	E2	c.202G>A	E68K
P005	*IL2RG*	E2	c.202G>A	E68K
P006	*IL2RG*	E2	c.252C>A	N84K
P007	*IL2RG*	I2	g.IVS2−15A>G	Predicted aberrant splicing
P008	*IL2RG*	I2	g.IVS2−15A>G	Predicted aberrant splicing
P011^e^	*IL2RG*	E3	c.310_311delinsG	H104fsX146
P012	*IL2RG*	E3	c.340G>T	G114C
P013^e^	*IL2RG*	E3	c.359dupA	K120fsX167
P014	*IL2RG*	E3	c.362delA	E121fsX146
P015	*IL2RG*	E3	c.365T>C	I122T
P016	*IL2RG*	E3	c.365T>C	I122T
P017^e^	*IL2RG*	E3	c.371T>C	L124P
P018	*IL2RG*	E3	c.376C>T	Q126X
P019^e^	*IL2RG*	E3	c.376C>T	Q126X
P020	*IL2RG*	E3	c.383T>C	F128S
P021	*IL2RG*	E3	c.386T>A	V129D
P022	*IL2RG*	E3	c.406_415del	R136fsX143
P023^e^	*IL2RG*	E3	c.421delC	Q141fsX146
P024	*IL2RG*	I3/E4 junction	g.IVS3−2A>T	Predicted aberrant splicing
P025^e^	*IL2RG*	E4	c.507delG	Q169fsX170
P026	*IL2RG*	E4	c.507delG	Q169fsX170
P027^e^	*IL2RG*	E4	c.562C>T	Q188X
P028	*IL2RG*	E4	c.562C>T	Q188X
P030	*IL2RG*	E5	c.658_659del	T220fsX227
P031	*IL2RG*	E5	c.664C>T	R222C
P032^e^	*IL2RG*	E5	c.670C>T	R224W
P033	*IL2RG*	E5	c.670C>T	R224W
P034	*IL2RG*	E5	c.676C>T	R226C
P036^e^	*IL2RG*	E5	c.677G>A	R226H
P037	*IL2RG*	E5	c.694G>C	G232R
P038	*IL2RG*	E5	c.709T>C	W237R
P039^e^	*IL2RG*	E5	c.711G>A	W237X
P040^e^	*IL2RG*	E5	c.722G>T	S241I
P041	*IL2RG*	E5	c.741delG	G247fsX272
P042	*IL2RG*	E5	c.741_742insG	E248fsX302
P044	*IL2RG*	E6	c.811G>T	G271X
P045^e^	*IL2RG*	E6	c.835delG	V279fsX293
P046	*IL2RG*	E6/I6 junction	c.854G>T	R285L
P047^e^	*IL2RG*	E6/I6 junction	c.854G>A^f^	Predicted aberrant splicing
				R285Q
P048^e^	*IL2RG*	E6/I6 junction	c.854G>A^f^	Predicted aberrant splicing
				R285Q
P049^e^	*IL2RG*	E6/I6 junction	c.854G>A^f^	Predicted aberrant splicing
				R285Q
P050^e^	*IL2RG*	E6/I6 junction	c.854G>A^f^	Predicted aberrant splicing
				R285Q
P051	*IL2RG*	E6/I6 junction	c.854G>A^f^	Predicted aberrant splicing
				R285Q
P052	*IL2RG*	E6/I6 junction	c.854G>A^f^	Predicted aberrant splicing
				R285Q
P053^e^	*IL2RG*	I6-I7	g.IVS6-72_IVS7-11del	Predicted exon 7 deletion
P055	*IL2RG*	I6/E7 junction	g.IVS6−2A>C	Predicted aberrant splicing
P056	*IL2RG*	I6	g.IVS6+3G>T	Predicted aberrant splicing
P057^e^	*IL2RG*	I6	g.IVS6+5G>A	Predicted aberrant splicing
P058^e^	*IL2RG*	I6	g.IVS6+5G>A	Predicted aberrant splicing
P059	*IL2RG*	E7	c.865C>T	R289X
P060	*IL2RG*	E8	c.929G>A	W310X
P062	*IL2RG*	E8	c.979_980delinsTT	E327L
P063a	*IL2RG*	E8	c.982C>T	R328X
P064	*ADA*	E7	c.646G>A	G216R
		E11	c.1018_1019del	K340fsX348
P065^b^	*DCLRE1C*	E1-E3	Gross deletion	Gross deletion
P066^b^	*DCLRE1C*	E1-E4	Gross deletion	Gross deletion
P068^e^	*DCLRE1C*	I3/E4 junction	c.IVS3−1G>T	I83-G102del
			Exon 4 skipped^a^	
		E8	c.632G>T	G211V
P069^e^	*IL7R*	E1	c.65G>T	S22I
		E2/I2 junction	g.IVS2+2T>A	Predicted aberrant splicing
P070^b,e^	*IL7R*	E5	c.562delC	L188X
P071^b^	*IL7R*	E5	c.616C>T	R206X
P072	*JAK3*	E2	c.115dupC	Q39fsX51
		E13	c.1744C>T	R582W
P073	*JAK3*	E3	c.307C>T	R103C
		E10	c.1333C>T	R445X
P074^e^	*JAK3*	E13	c.1763A>C	H588P
P075^b,e^	*JAK3*	I14	g.IVS14−11G>A	638_639insPPX
			c.1914_1915insCCCCCTTAG^a^	
P076^c^	*JAK3*	E16	c.2062A>T	I688F
P077	*RAG1*	E2	c.994C>T	R332X
		E2	c.3074dupT	L1025fsX1064
P078^e^	*RAG1*	E2	c.1178delG	G393fsX402
		E2	c.2095C>T	R699W
P079	*RAG1*	E2	c.1328G>A	R443K
		E2	c.2486_2490del	R829fsX869
P080	*RAG1*	E2	c.1681C>T	R561C
		E2	c.2561G>A	G854D
P081^d^	*RAG1*	E2	c.2005G>A	E669K
P083	*RAG1*	E2	c.2324T>A	L775Q
		E2	c.2918G>A	R973H
P084^e^	*RAG2*	E1/I1 junction	c.-28G>C	Predicted aberrant splicing
		E2	c.358delG	V120fsX130
P085^b^	*RAG2*	E2	c.104G>T	G35V
P086^b^	*RAG2*	E2	c.104G>T	G35V
P087	*RAG2*	E2	c.104G>T	G35V
		E2	c.475C>T	R159C
P088^b^	*RAG2*	E2	c.218G>A	R73H
P089	*RAG2*	E2	c.442C>T	R148X
		E2	c.685C>T	R229W
P091	*RFXANK*	E3/I3 junction	g.IVS3+1delG	Predicted aberrant splicing
		E5	c.299_300del	Q100fsX113
P092^b^	*RFXANK*	E5	c.299_300del	Q100fsX113

*ADA, adenosine deaminase; DCLRE1C, DNA cross-link repair enzyme 1C; IL2RG, interleukin-2 receptor subunit gamma; IL7R, interleukin-7 receptor subunit alpha; JAK3, Janus kinase 3; RAG, recombinase activating genes; RFXANK, regulatory factor X-associated ankyrin-containing protein*.

*^a^From RT-PCR results*.

*^b^Homozygous mutations*.

*^c^P076 was a B+NK− patient with hypogammaglobulinemia (IgG 1.45 g/L, IgA 0.23 g/L, and IgM 0.26 g/L) whose mother was a heterozygous carrier*.

*^d^Only one mutation was found*.

*^e^Patients reported in our previous study (13)*.

*^f^Previous study reported 854G>A may cause R285Q or skipping of exon 6 (27). P004a and P004b and P063a and P063b (Table SE1 in Supplementary Material) were from the same kindred*.

Genetic mutations in all SCID genes were not evenly distributed, and two mutations were seen three or more times in unrelated patients. c.854G>A mutation was seen in six unrelated patients with *IL2RG* mutation. c.104G>T mutation was observed in three unrelated patients with *RAG2* mutation.

Twenty-two C>T or G>A mutations within CpG dinucleotides were documented (8 *IL2RG* mutations, 5 *RAG1* mutations, 4 *RAG2* mutations, 3 *JAK3* mutations, 1 *IL7R* mutation, and 1 *ADA* mutation). These mutations accounted for 25% of all mutations and were involved in 31 patients (18 in *IL2RG*, 5 in *RAG1*, 3 in *RAG2*, 2 in *JAK3*, 1 in *IL7R*, and 1 in *ADA*).

There were 29 unreported mutations identified in our patients, including 23 *IL2RG* mutations, 3 *RAG1* mutations, 1 *JAK3* mutation, 1 *RAG2* mutation, and 1 *RFXANK* mutations (Table SE3 in Supplementary Material). Effects of these unreported mutations on protein functions were predicted by multiple tools and are shown in Table SE3 in Supplementary Material.

### Characteristics of Patients That Fulfilled Selection Criteria (*n* = 83)

Characteristics of patients included in our study (*n* = 131) are shown in Tables 2–4. For patients that fulfilled selection criteria (*n* = 83), 88.0% were male (*n* = 73) and 75.9% were Chinese (*n* = 63). The median AP was 2 months (0.1–6 months), AD 4 months (0.5–18 months), and time to diagnosis 2 months (0–14 months). Twenty-nine patients (34.9%) had a family history of early infant death (FH), among them one patient had a family history of SCID and one patient had a family history of PID. Parental consanguinity was present in four kindreds. The median ALC was 1.05 × 10^9^/L (0.134−52.2 × 10^9^/L, *n* = 70) with 88.6% below 3 × 10^9^/L (*n* = 62). The major immunophenotype was B+ SCID (*n* = 51) (Tables 2 and 3).

**Table 2:** Characteristics of patients included in our study (*n* = 131) at SCID diagnosis.

	With genetic diagnosis	Without genetic diagnosis

	*n* = 83	*n* = 48
**Gender**	**Number (%)**	**Number (%)**
Male	73 (88.0)	33 (68.8)
Female	10 (12.0)	15 (31.3)
**Ethnicity**	**Number (%)**	**Number (%)**
Chinese	63 (75.9)	30 (62.5)
Southeast Asian	12 (14.5)	4 (8.3)
Indonesian	1 (1.2)	0 (0)
Malay	3 (3.6)	3 (6.3)
Philippino	1 (1.2)	0 (0)
Thai	5 (6.0)	1 (2.1)
Vietnamese	2 (2.4)	0 (0)
Indian	2 (2.4)	9 (18.8)
Algerian	5 (6.0)	0 (0)
Arabian	1 (1.2)	3 (6.3)
Australian	0 (0)	1 (2.1)
Korean	0 (0)	1 (2.1)
**Positive family history**	**Number (%)**	**Number (%)**
Early infant death	29 (34.9)	13 (27.1)
Consanguinity	4 (4.8)	1 (2.1)
**Age in months**	**Median (range)**	**Median (range)**
Age at presentation	2 (0.1–6)	2 (0–19)
Age at diagnosis	4 (0.5–18)	4 (0.1–27)
Time to diagnosis	2 (0–14)	2 (0–16)
**SCID phenotype**	**Number (%)**	**Number (%)**
B+	51 (61.4)	18 (37.5)
B−	15 (18.1)	24 (50.0)
Others	17 (20.5)^a^	6 (12.5)^b^
	**Median (range)**	**Median (range)**
Absolute lymphocyte count (10^9^/L)	1.05 (0.134–52.2)^c^	0.77 (0.09–13.46)^d^

*^a^Maternal engraftment (*n* = 1), unknown (*n* = 16)*.

*^b^Unknown (*n* = 6)*.

*^c^In 70 patients*.

*^d^In 44 patients*.

**Table 3:** Lymphocyte subset for patients included in our study (*n* = 131).

Patient	Mutation gene	ALC (×10^9^/L)	CD3^**+**^ cells/μL (%)	CD19^**+**^ cells/μL (%)	CD16/56^**+**^ cells/μL (%)
**B+ SCID**					
P006	*IL2RG*	0.4	9.2 (2.3)	385.6 (96.4)	2.4 (0.6)
P008	*IL2RG*	0.95	0 (0)	931 (98)	9.5 (1)
P011	*IL2RG*	1.16	3.48 (0.3)	972 (83.8)	2.3 (0.2)
P013	*IL2RG*	0.31	0 (0)	270 (87)	0 (0)
P014	*IL2RG*	2.93	468 (16)	2,344 (80)	58.6 (2)
P015	*IL2RG*	0.51	0 (0)	459 (90)	10.2 (2)
P017	*IL2RG*	0.7	0.7 (0.1)	663 (94.7)	36.4 (5.2)
P018	*IL2RG*	1	20 (2)	890 (89)	0 (0)
P019	*IL2RG*	2.63	26.3 (1)	2,525 (96)	26.3 (1)
P020	*IL2RG*	0.38	22.8 (6)	345.8 (91)	0 (0)
P021^b^	*IL2RG*	1.66	596 (35.9)	1,061 (63.9)	2 (0.12)
P022	*IL2RG*	0.33	7.59 (2.3)	313.5 (95)	6.6 (2)
P024^b^	*IL2RG*	3.43	504 (14.7)	2,867 (83.6)	58.3 (1.7)
P025	*IL2RG*	1.4	0 (0)	1,302 (93)	42 (3)
P026	*IL2RG*	0.33	16.5 (5)	290.4 (88)	9.9 (3)
P027	*IL2RG*	1.1	5.5 (0.5)	1,022 (92.9)	14.3 (1.3)
P028	*IL2RG*	5	4,600 (92)	300 (6)	100 (2)
P030	*IL2RG*	0.94	16 (1.7)	620.4 (66)	192.7 (20.5)
P031	*IL2RG*	5.1	948 (18.6)	3,042 (59.7)	928.7 (18.2)
P032	*IL2RG*	1.1	11 (1)	979 (89)	110 (10)
P033	*IL2RG*	0.99	5 (0.5)	585.1 (59.1)	17.8 (1.8)
P034	*IL2RG*	1.34	0 (0)	1,112 (83)	160.8 (12)
P036	*IL2RG*	1.11	11.1 (1)	455.1 (41)	577 (52)
P038	*IL2RG*	0.6	240 (40)	324 (54)	33 (5.5)
P039	*IL2RG*	1.72	0 (0)	1,170 (68)	498.8 (29)
P041	*IL2RG*	0.62	12.4 (2)	545.6 (88)	37.2 (6)
P044	*IL2RG*	0.9	0 (0)	846 (94)	18 (2)
P045	*IL2RG*	1.84	0 (0)	1,748 (95)	73.6 (4)
P047	*IL2RG*	1.41	155.1 (11)	1,197 (84.9)	18.3 (1.3)
P048	*IL2RG*	4.94	0 (0)	4,841 (98)	98.8 (2)
P049	*IL2RG*	2.1	0 (0)	2,079 (99)	0 (0)
P050	*IL2RG*	0.53	5.3 (1)	424 (80)	21.2 (4)
P051	*IL2RG*	1.1	0 (0)	1,067 (97)	11 (1)
P052	*IL2RG*	1.86	223.2 (12)	1,600 (86)	0 (0)
P053	*IL2RG*	1	52 (5.2)	892 (89.2)	N/A (N/A)
P055	*IL2RG*	0.9	9 (1)	828 (92)	9 (1)
P056	*IL2RG*	1.3	1 (0.08)	1,282 (98.6)	9.6 (0.74)
P058	*IL2RG*	1.3	0 (0)	611 (47)	18.2 (1.4)
P059	*IL2RG*	1.18	0 (0)	1,133 (96)	35.4 (3)
P060	*IL2RG*	1.5	45 (3)	1,350 (90)	30 (2)
P063a	*IL2RG*	0.62	61.4 (9.9)	484.2 (78.1)	46.5 (7.5)
P069	*IL7R*	1.89	183.3 (9.7)	1,111 (58.8)	565.1 (29.9)
P070	*IL7R*	1.21	147.6 (12.2)	756.3 (62.5)	410.2 (33.9)
P071	*IL7R*	0.785	1.6 (0.2)	148.4 (18.9)	433.3 (55.2)
P072	*JAK3*	2.52	1,738 (69)	730.8 (29)	0 (0)
P073	*JAK3*	0.47	4.7 (1)	437.1 (93)	N/A (N/A)
P074	*JAK3*	0.35	91 (26)	196 (56)	N/A (N/A)
P075	*JAK3*	0.49	6.4 (1.3)	266.1 (54.3)	19.6 (4)
P076	*JAK3*	1.5	12.6 (0.84)	1,377 (91.77)	54.5 (3.63)
P078	*RAG1*	7.64	3,965 (51.9)	267.4 (3.5)	3,705 (48.5)
P091	*RFXANK*	1.59	624.9 (39.3)	936.5 (58.9)	47.7 (3)
P094	N/A	2.06	195.7 (9.5)	1,788 (86.8)	76.2 (3.7)
P098	N/A	1.23	764.2 (62.13)	156.9 (12.76)	263.1 (21.39)
P099	N/A	13.46	9,826 (73)	1,346 (10)	1,750 (13)
P109	N/A	0.88	295.7 (33.6)	460.2 (52.3)	89.5 (10.2)
P110	N/A	2.06	68 (3.3)	1,593 (77.3)	345.7 (16.8)
P111	N/A	1.46	18.3 (1.25)	1,387 (95)	6.6 (0.45)
P112	N/A	2.42	217.8 (9)	1,500 (62)	532.4 (22)
P115	N/A	1.84	18.4 (1)	1,472 (80)	294.4 (16)
P116	N/A	1.3	26 (2)	1,040 (80)	130 (10)
P117	N/A	2.1	396.9 (18.9)	573.3 (27.3)	136.5 (6.5)
P120	N/A	1.09	21.8 (2)	1,030 (94.5)	3.3 (0.3)
P121	N/A	0.5	20 (4)	245 (49)	205 (41)
P122	N/A	0.41	32.8 (8)	278.8 (68)	86.1 (21)
P123^b^	N/A	1.28	65.3 (5.1)	833.3 (65.1)	381.4 (29.8)
P124	N/A	0.8	15.2 (1.9)	724 (90.5)	43.2 (5.4)
P126	N/A	0.84	342.7 (40.8)	207.5 (24.7)	197.4 (23.5)
P128	N/A	3.5	2,485 (71)	455 (13)	455 (13)
P137	N/A	0.9	9.9 (1.1)	136.8 (15.2)	419.4 (46.6)
**B− SCID**					
P001	*IL2RG*	0.14	0 (0)	70 (50)	1.4 (1)
P002	*IL2RG*	0.67	636.5 (95)	13.4 (2)	0 (0)
P003	*IL2RG*	3.6	3,456 (96)	0 (0)	N/A (N/A)
P005	*IL2RG*	0.18	7.2 (4)	3.6 (2)	145.8 (81)
P064	*ADA*	0.21	4 (1.9)	1 (0.48)	16.8 (8)
P065	*DCLRE1C*	0.65	110.5 (17)	26 (4)	78 (12)
P066	*DCLRE1C*	1.2	36 (3)	24 (2)	1,080 (90)
P068	*DCLRE1C*	0.72	7.2 (1)	0.72 (0.1)	672.5 (93.4)
P079	*RAG1*	0.96	144 (15)	1.9 (0.2)	796.8 (83)
P080	*RAG1*	0.134	132 (98.6)	0.04 (0.03)	1.5 (1.1)
P083	*RAG1*	0.34	80.6 (23.7)	3.1 (0.9)	190.1 (55.9)
P084	*RAG2*	0.74	7.4 (1)	7.4 (1)	666 (90)
P087	*RAG2*	2.55	2.6 (0.1)	132.6 (5.2)	2,020 (79.2)
P088	*RAG2*	28.36	26,772 (94.4)	0 (0)	623.9 (2.2)
P092	*RFXANK*	1.019	276.1 (27.1)	19.4 (1.9)	25.5 (2.5)
P093	N/A	0.31	N/A (N/A)	2 (0.65)	120.9 (39)
P095	N/A	3.38	3,191 (94.4)	33.8 (1)	33.8 (1)
P097	N/A	2.49	2,366 (95)	18.9 (0.76)	49.8 (2)
P100	N/A	0.489	477.8 (97.7)	2.9 (0.6)	4.9 (1)
P101	N/A	0.242	15 (6.19)	2.9 (1.2)	206.4 (85.3)
P102	N/A	0.09	41.1 (45.7)	1.4 (1.6)	19.5 (21.7)
P103	N/A	1.8	1,499 (83.3)	3.6 (0.2)	257.4 (14.3)
P104	N/A	0.138	26.2 (19)	1.4 (1)	93.8 (68)
P105	N/A	0.72	144 (20)	0.72 (0.1)	537.1 (74.6)
P106	N/A	0.42	408.2 (97.2)	1.3 (0.3)	4.6 (1.1)
P107	N/A	0.65	76.7 (11.8)	29.3 (4.5)	490.8 (75.5)
P108	N/A	0.8	40 (5)	14.4 (1.8)	656 (82)
P114	N/A	0.59	11.8 (2)	15.9 (2.7)	472 (80)
P118	N/A	0.19	0 (0)	39.9 (21)	0.38 (0.2)
P125	N/A	0.84	579.6 (69)	100.8 (12)	134.4 (16)
P127	N/A	0.53	312.2 (58.9)	73.1 (13.8)	19.6 (3.7)
P130	N/A	0.29	70.8 (24.4)	45.8 (15.8)	150.8 (52)
P131	N/A	0.8	16 (2)	0 (0)	768 (96)
P132	N/A	0.74	583.1 (78.8)	17.8 (2.4)	96.2 (13)
P134	N/A	0.7	539 (77)	0 (0)	1.1 (0.16)
P135	N/A	1.03	20.6 (2)	30.9 (3)	875.5 (85)
P136	N/A	0.28	254.8 (91)	2.8 (1)	19.6 (7)
P138	N/A	0.22	72.6 (33)	2.2 (1)	129.8 (59)
P139	N/A	0.1	42.8 (42.8)	4.1 (4.1)	50 (50)

**Others**					

**Maternal engraftment**					
P077	*RAG1*	52.23	49,619 (95)	0 (0)	2,089 (4)
**Unknown**					
P004a	*IL2RG*	0.64	0 (0)	N/A^a^	N/A^a^
P004b	*IL2RG*	N/A	N/A (2)	N/A (85)	N/A (10)
P007	*IL2RG*	N/A	N/A (0.2)	N/A (86.7)	N/A (6.7)
P012	*IL2RG*	N/A	N/A (2)	N/A (95)	N/A (0)
P016	*IL2RG*	N/A	N/A (N/A)	N/A (N/A)	N/A (N/A)
P023	*IL2RG*	0.5	N/A (N/A)	N/A (N/A)	N/A (N/A)
P037	*IL2RG*	1.6	N/A (N/A)	N/A (N/A)	N/A (N/A)
P040	*IL2RG*	N/A	N/A (16)	N/A (82)	N/A (0)
P042	*IL2RG*	N/A	N/A (2)	N/A (95)	N/A (1)
P046	*IL2RG*	N/A	N/A (0)	N/A (93)	N/A (2)
P057	*IL2RG*	N/A	N/A (0)	N/A (89)	N/A (0)
P062	*IL2RG*	5.4	N/A (N/A)	N/A (N/A)	N/A (N/A)
P081	*RAG1*	N/A	N/A (1.5)	N/A (0.52)	N/A (74.1)
P085	*RAG2*	N/A	N/A (13)	N/A (0.1)	N/A (24)
P086	*RAG2*	N/A	N/A (0.67)	N/A (0)	N/A (76)
P089	*RAG2*	N/A	N/A (N/A)	N/A (N/A)	N/A (N/A)
P096	N/A	0.26	N/A (N/A)	N/A (N/A)	N/A (N/A)
P113	N/A	N/A	N/A (1)	N/A (87)	N/A (4)
P119	N/A	N/A	N/A (3)	N/A (67.5)	N/A (25.4)
P129	N/A	0.22	N/A (N/A)	N/A (N/A)	N/A (N/A)
P133	N/A	0.6	N/A (N/A)	N/A (N/A)	N/A (N/A)
P140	N/A	N/A	N/A (2.8)	N/A (0.6)	N/A (90)

*B+ SCID was defined as having ≥134 CD19^+^ cells/μL and B− SCID was defined as having <134 CD19^+^ cells/μL. ALC, absolute lymphocyte count; N/A, not available*.

*^a^Medical record documented as “raised”*.

*^b^ALC was not provided, derived by summation of CD3^+^ cells, CD19^+^ cells, and CD16/56^+^ cells, in P024 the ALC was documented as 2.7 × 10^9^/L in separate test*.

50.6% of patients presented with chronic diarrhea (*n* = 42) and 60.2% of patients recurrent infections (*n* = 50). The commonest site of infection was the respiratory system (*n* = 61), followed by gastrointestinal system (*n* = 42). 47.0% of infections were severe (*n* = 39).

Fifty patients developed opportunistic infection (60.2%). The commonest opportunistic infection was candidiasis (*n* = 27), followed by BCG infection (*n* = 19) and viral infection (*n* = 9). The median age for candidiasis documented was 3 months, the median age for BCG infection was 4 months, and the median age for CMV infection was 2.25 months.

For patients included in our study, clinical features were compared between those with (*n* = 83) and without genetic diagnosis (*n* = 48). Patients without genetic diagnosis had higher frequency of FTT (33.3 versus 15.7%, *p* = 0.0189) and CMV infections (25.0 versus 9.6%, *p* = 0.0185) (Table 4).

**Table 4:** Clinical features of patients included in our study (*n* = 131).

	With genetic diagnosis	Without genetic diagnosis
	*n* = 83	*n* = 48

	Number (%)	Number (%)
**Classical SCID triad**		
Failure to thrive	13 (15.7)	16 (33.3)^a^
Chronic diarrhea	42 (50.6)	27 (56.3)
Recurrent infections	50 (60.2)	28 (58.3)
**Infection by systems**		
Respiratory infection	61 (73.5)^c^	34 (70.8)^d^
Non-bacillus Calmette–Guérin (BCG) skin and soft tissue infection	7 (8.4)	11 (22.9)
Gastrointestinal infection	42 (50.6)	23 (48.9)
Urogenital infection	2 (2.4)	1 (2.1)
Musculoskeletal infection	3 (3.6)	1 (2.1)
Central nervous system infection	1 (1.2)	1 (2.1)
Sepsis	18 (21.7)	8 (16.7)
**Severe infections**	39 (47.0)	26 (54.2)
Intensive care unit admission	24 (28.9)	14 (29.2)
Life support	26 (31.3)	14 (29.2)
Intubation and ventilation	21 (25.3)	8 (16.7)
Resuscitation and/or inotrope support	5 (6.0)	9 (18.8)
Life-threatening complication	40 (48.2)	18 (37.5)
Sepsis	18 (21.7)	8 (16.7)
Respiratory distress/failure	26 (31.3)	13 (27.1)
Acute heart failure	2 (2.4)	1 (1.2)
**Opportunistic infections**	50 (60.2)	28 (58.3)
Bacterial	8 (9.6)	1 (2.1)
*Pseudomonas aeruginosa*	6 (7.2)	0 (0)
*Acinetobacter baumanii*	4 (4.8)	1 (2.1)
Viral	9 (10.8)	12 (25.0)
Cytomegalovirus (CMV)	8 (9.6)^e^	12 (25.0)^b^
Herpes zoster	1 (1.2)	0 (0)
Bacillus Calmette–Guérin (BCG) infection	19 (22.9)^f^	8 (16.7)
Local	8 (9.6)	2 (4.2)
Regional	2 (2.4)	1 (2.1)
Disseminated	9 (10.8)	5 (10.4)
Candidiasis	27 (32.5)^g^	16 (33.3)
Persistent oral thrush	22 (26.5)	10 (20.8)
Gastrointestinal tract infection	3 (3.6)	2 (4.2)
Candidemia	2 (2.4)	4 (8.3)
Fungal	3 (3.6)	2 (4.2)
*Pneumocystis jiroveci*	2 (2.4)	1 (2.1)
Aspergillosis	1 (1.2)	1 (2.1)
**Hepatosplenomegaly**	12 (14.5)	9 (18.8)

*^a^p = 0.0189*.

*^b^p = 0.0185*.

*^c^A total of 53 patients with genetic diagnosis had pneumonia (63.9%)*.

*^d^A total of 32 patients without genetic diagnosis had pneumonia (66.7%)*.

*^e^The median age for CMV infection documented was 2.25 months (*n* = 8)*.

*^f^The median age for BCG infection documented was 4 months (*n* = 14)*.

*^g^The median age for candidiasis documented was 3 months (*n* = 25)*.

For patients with documented ALC (*n* = 114), 107 of them (93.9%) had at least one of the following four clinical features: FH, candidiasis, BCG infection, and ALC below 3 × 10^9^/L. 65 of them (57.0%) had at least two of the four clinical features mentioned.

### FH and Pneumonia Were Associated with Earlier AP

Factors that were found to significantly affect AP, AD, and time to diagnosis are shown in Tables 5 and 6.

**Table 5:** Univariate analysis of features that affect age at presentation (AP), age at diagnosis, and time to diagnosis in patients fulfilled selection criteria (*n* = 83).

Features	Median AP (months) when		Difference in months (Group A–Group B)	*p*-Value
	Feature present (Group A)	Feature absent (Group B)		
FH	1	2	−1	0.002
Candidiasis	2	2	0	0.664
Bacillus Calmette–Guérin (BCG)	2	2	0	0.291
CMV	1	2	−1	0.280
FTT	2	2	0	0.954
Chronic diarrhea	2	2	0	0.778
Recurrent infections	2	3	−1	0.008
Severe infections	2	2	0	0.813
Pneumonia	2	3	−1	0.003
Hepatosplenomegaly	2.25	2	0.25	0.347
X-linked SCID	2	2	0	0.057
Low ALC^a^	2	2.25	−0.25	0.771

**Features**	**Median age at diagnosis (months) when**		**Difference in months (Group C–Group D)**	***p*-Value**
	**Feature present (Group C)**	**Feature absent (Group D)**		

FH	3	5	−2	0.008
Candidiasis	6	4	2	0.008
BCG	6	4	2	0.005
CMV	3	4	−1	0.025
FTT	7	4	3	0.038
Chronic diarrhea	4	4.5	−0.5	0.949
Recurrent infections	5	4	1	0.241
Severe infections	4	5	−1	0.476
Pneumonia	4	5	−1	0.111
Hepatosplenomegaly	4	4	0	0.544
X-linked SCID	4	3.5	0.5	0.689
Low ALC^a^	4	6.5	−2.5	0.086

**Features**	**Median time to diagnosis (months) when**		**Difference in months (Group E–Group F)**	***p*-Value**
	**Feature present (Group E)**	**Feature absent (Group F)**		

FH	2	2	0	0.494
Candidiasis	2.5	1.95	0.55	0.003
BCG	3	2	1	0.052
CMV	1.25	2	−0.75	0.155
FTT	4	2	2	0.104
Chronic diarrhea	1.15	2	−0.85	0.617
Recurrent infections	2.5	1	1.5	<0.001
Severe infections	2	2	0	0.565
Pneumonia	2	1.75	0.25	0.382
Hepatosplenomegaly	1.5	2	−0.5	0.217
X-linked SCID	2	2	0	0.569
Low ALC^a^	2	4.5	−2.5	0.124

*^a^Defined as ALC below 3 × 10^9^/L*.

*FH, family history of early infant death; CMV, cytomegalovirus infection; FTT, failure to thrive, ALC, absolute lymphocyte count*.

**Table 6:** Multivariate linear regression of features that affect age at presentation (AP), age at diagnosis (AD), and time to diagnosis in patients fulfilled selection criteria (*n* = 83).

Features	Regression coefficient (months)	*p*-Value	95% CI
**AP**			
FH	−0.884	0.005	−1.499 to −0.269
Recurrent infections	−0.541	0.086	−1.161 to 0.078
Pneumonia	−0.863	0.009	−1.504 to −0.221
**AD**			
FH	−1.86	0.007	−3.189 to −0.529
Candidiasis	2.21	0.002	0.858 to 3.555
Bacillus Calmette–Guérin	1.11	0.141	−0.375 to 2.595
CMV	−1.58	0.147	−3.727 to 0.569
FTT	1.15	0.190	−0.584 to 2.886
**Time to diagnosis**			
Candidiasis	1.511	0.018	0.267 to 2.754
Recurrent infections	1.845	0.003	0.655 to 3.036

*FH, family history of early infant death; CMV, cytomegalovirus infection; FTT, failure to thrive; 95% CI, 95% confidence interval*.

In univariate analysis, FH, pneumonia, and recurrent infections were associated with earlier AP (FH by 1 month, *p* = 0.002; pneumonia by 1 month, *p* = 0.003; recurrent infections by 1 month, *p* = 0.008). Upon multivariate analysis, only FH and pneumonia were associated with earlier AP (FH by 0.884 month, *p* = 0.005; pneumonia by 0.863 month, *p* = 0.009).

### FH Was Associated with Earlier AD

In univariate analysis, FH and CMV infections were associated with an earlier AD (FH by 2 months, *p* = 0.008; CMV by 1 month, *p* = 0.025). Upon multivariate analysis, only FH was associated with earlier AD (by 1.86 months, *p* = 0.007).

### Candidiasis and Opportunistic Infections Were Associated with Later AD

In univariate analysis, candidiasis, FTT, opportunistic infections, and BCG infection were associated with a later AD (candidiasis by 2 months, *p* = 0.008; FTT by 3 months, *p* = 0.038; opportunistic infections by 1 month, *p* = 0.018; BCG by 2 months, *p* = 0.005). Upon multivariate analysis, only candidiasis was associated with later AD (by 2.21 months, *p* = 0.002).

### Candidiasis, Opportunistic Infections, and Recurrent Infections Were Associated with Longer Time to Diagnosis

Candidiasis, opportunistic infections, and recurrent infections were shown to be associated with longer time to diagnosis (candidiasis by 0.55 month, *p* = 0.003; opportunistic infections by 1 month, *p* = 0.005; recurrent infections by 1.5 months, *p* < 0.001). Upon multivariate analysis, both candidiasis and recurrent infections were associated with longer time to diagnosis (candidiasis by 1.51 months, *p* = 0.018; recurrent infections by 1.85 months, *p* = 0.003).

### Other Features Were Not Significantly Associated with AD and Time to Diagnosis

Analysis of chronic diarrhea, pneumonia, hepatosplenomegaly, severe infections, and lymphopenia revealed no association with AD and time to diagnosis. There was no difference between X-linked and autosomal recessive forms of SCID in AP, AD, and time to diagnosis.

## Discussion

We found family history of early infant death was associated with earlier AP and earlier AD but not shorter time to diagnosis. Therefore, the earlier AD could be due to the heightened alertness of family with such history so that medical attention was sought earlier, rather than prompting clinicians in making quicker SCID diagnosis. The association between positive family histories and earlier AD was reported by studies in USA and France (10, 28), but they did not investigate whether the positive family history shortened the time to diagnosis. Moreover, they did not investigate whether presence of family history of early infant death alone is associated with an earlier AD. Previous studies reported 16–60% of patients with positive family histories compared to that of 32% in our study; however, the definition of family history differs between studies (Table 7). Our findings suggested that the family history of early infant death was valuable in alerting families but not clinicians who failed to recognize this clue as the time to diagnosis remained the same regardless of the presence of family history of early infant death.

**Table 7:** Comparison with previous SCID studies.

Cohort	*n*	Origin	Duration	AP (months)	AD (months)	Genotype (%)			ALC (×10^9^/L)	Present in cases (%)		
						IL2RG	Other	Unknown		FH	Candidiasis	BCG infection
Our study	131	Asia	2005–2016	2 (0–19)^a^	4 (0.1–27)^a^	57 (44)	26 (20)	48 (37)	0.975 (0.09–52.20)^a^	42/131 (32)^f^	43/131 (33)	27/131 (21)^e^
Stephan et al. (28)	117	France	1970–1992	3 (0–19)^c^	4.6 (0–27)^c^	0 (0)	0 (0)	117 (100)	1.608 (0–30)^c^	70/117 (60)	33/117 (28)	10/28 (36)^e^
Mazzucchelli et al. (29)	70	Brazil	1996–2011	2 (0–19)^a^	8 (0–22)^a^	0 (0)	0 (0)	70 (100)	N/A	19/70 (27)^g^	29/64 (45)	39/69 (57)^e^
Yeganeh et al. (30)	40	Iran	1999–2007	2.26 (±0.43)^b^	5 (±0.67)^b^	0 (0)	0 (0)	40 (100)	1.26^a^	20/40 (50)^h^	23/40 (58)	18/40 (45)^e^
Saleem et al. (31)	13	Pakistan	2006–2011	N/A	5 (1.23–8.93)^a^	0 (0)	0 (0)	13 (100)	0.41 (0.17–2.28)^a^	7/13 (54)^f^	N/A	N/A^e^

McWilliams et al. (10)	172	USA	1982–2013	N/A	4.87 (0–18)^b^	77 (45)	91 (53)	4 (2)	0.43 (±1.28)^b^	63/172 (37)^h^	74/172 (43)	2/172 (1)
Dvorak et al. (32)	50	North America	2010–2012	N/A	1.13 (0–10.13)^d^	20 (40)	27 (54)	3 (6)	1.22 (0.02–10.72)^a^	12/50 (24)^i^	8/50 (16)	0/0 (0)
Rozmus et al. (33)	40	Canada	2004–2010	N/A	4.2 (0–19.4)^c^	4 (10)	16 (40)	20 (50)	1.13 (0.05–14.04)^a^	20/39 (51)^j^	8/39 (21)	0/0 (0)

*AP, age at presentation; AD, age at diagnosis; ALC, absolute lymphocyte count; FH, positive family history*.

*^a^Median (range)*.

*^b^Mean (SD)*.

*^c^Mean (range)*.

*^d^Typical SCID (*n* = 37) only*.

*^e^With universal BCG vaccination at birth*.

*^f^FH of early infant death*.

*^g^Suggestive or confirmed FH of SCID*.

*^h^FH of early infant death due to infection or known SCID*.

*^i^FH of immunodeficiency*.

*^j^FH of SCID and/or infant death and consanguinity*.

We found candidiasis was associated with later AD and longer time to diagnosis. The median age of candidiasis documented was 3 months, and the median AD of SCID for patients with candidiasis was 6 months. Therefore, clinicians required 3 months to diagnose SCID after candidiasis was first documented. This suggested that candidiasis was an overlooked feature by clinicians in Asia. Other studies reported similar percentage at candidiasis in SCID patients but no report of association between candidiasis and AD (Table 7). Although oral candidiasis is relatively common in infants under 6 months old; however, persistent, recurrent, or invasive candidiasis warrants investigation for underlying immunodeficiencies in particular SCID (15). Our finding suggested that candidiasis may be useful as a clue for earlier diagnosis since the median age of candidiasis documented was 3 months, which was earlier than the optimal time for HSCT at 3.5 months (9, 11).

We were surprised to find that BCG infection was not associated with AD and time to diagnosis. This could be due to the relatively low frequency of patients with BCG infections (21%) identified in our study, which was at a lower frequency when compared to that of 45–57% reported previously (6, 29, 30). The population coverage, immunization schedules, and virulence of BCG in countries and regions included in our study were comparable to that in Brazil and Iran (Table SE5 in Supplementary Material) (29, 34–39); therefore, the above factors of BCG policies could not account for the discrepancy in the frequency of BCG complications between our study and that from Brazil and Iran. In addition, the onset of BCG complication in our study at 4 months old was comparable to that in Brazil at 3.7 months old (29). The median AD of SCID in our study was 4 months, which was earlier than the 8 months in Brazil and 5 months in Iran, suggesting that the lower frequency of BCG infections in our study (21%) than that in Brazil (57%) and Iran (45%) (Table 7) could be due to earlier diagnosis of SCID in our study.

The median age for BCG infection documented in our patients was 4 months, which was beyond the optimal time for HSCT. Our findings were in line with a previous report in which 74% of 349 BCG-vaccinated SCID patients developed BCG infection at or after 4 months of age (6). Therefore, despite BCG infections being useful clinical features of SCID as SCID patients have approximately 400-fold increase in risk of having localized BCG complication and 33,000-fold increase in risk of having disseminated complications (6), noticing BCG infection had little value in alerting clinicians to make a timely diagnosis of SCID for optimal HSCT, which should be before 3.5 months (9, 11).

We found that opportunistic infections were associated with later AD, while recurrent infections and opportunistic infections were associated with longer time to diagnosis. Therefore, such clinical features were likely the consequences of delay in the diagnosis of SCID, reflecting that clinicians in Asia were unable to recognize these as SCID features.

We found that pneumonia was associated with an earlier AP but did not affect AD and time to diagnosis. Therefore, parents may perceive pneumonia as a severe medical condition and then brought their children to seek medical care earlier. However, pneumonia also commonly affects children without SCID in Asia (40–42), and clinicians are not alerted to the possible diagnosis of SCID.

Chronic diarrhea, severe infections, and ALC below 3 × 10^9^/L were not associated with AD and time to diagnosis, likely due to the distributions of the AD and the time to diagnosis that were quite wide in patients with these features (Figure S1 in Supplementary Material). In addition, chronic diarrhea is common in Southeast Asia and Western Pacific region (40); thus, it may not be a useful differentiating feature for patients with SCID as compared to those without. CMV infections did not affect AD and time to diagnosis as they were documented in small number of patients in our study (*n* = 8). The low rate of documented CMV infections may be due to the lack of diagnostic capacity (43, 44).

This study presented the largest collection of SCID patients in China and Southeast Asia with 147 patients, including 94 SCID patients with genetic diagnosis. The median AD was 4 months, which was comparable to other cohorts in the world, given no newborn screening of TREC was performed (Table 7) (10, 28–33); however, it was later than the optimal time for HSCT.

The commonest SCID gene found to be mutated in our patients was *IL2RG* because of the low consanguinity rate in our population (45) as well as near absence of newborn screening in Asia. Mutations in *IL2RG* were unevenly distributed. Exons 3 and 5 of *IL2RG* were common sites for mutation, accounting for 45% of all *IL2RG* mutations (Table 1; Table SE1 in Supplementary Material) and 48% of all unreported *IL2RG* mutations (Table SE3 in Supplementary Material), which was comparable with previous study (46). Five mutation hotspots, namely cDNA 670, 676, 677, 854, and 865, were identified previously and accounted for 29% of all *IL2RG* mutations in one study (46). Mutations in these hotspots collectively accounted for 27% of *IL2RG* mutations in our study. Majority of the mutations in these hotspots involved either C>T or G>A mutations in CpG dinucleotides. The mutation frequency of the C nucleotides in CpGs is 10–50 times higher compared to any other bases (47). This is commonly thought to be due to the methylation and subsequently deamination of cytosine to form thymidine in CpG (48, 49). Apart from the mentioned hotspots, we identified 16 additional point mutations in all SCID genes involving such mechanism, suggesting that cytosine methylation and deamination to thymidine in CpG dinucleotide is a relatively common mechanism causing mutations in SCID genes.

Four patients with mutations in *IL2RG* were classified as having B− SCID with CD19^+^ B cells ranging from 0 to 70/μL (Table 3). The four patients had typical SCID presentations (Table SE4 in Supplementary Material). One patient was screened for *DCLRE1C, RAG1*, and *RAG2* due to his B− phenotype, but no mutation found. Patients suffering from X-linked SCID but with T-B− phenotype have been described previously (28). Patients with documented *IL2RG* mutations but with T-B+NK+ phenotype were also described previously (50–52). Many possible mechanisms can lead to atypical SCID immunophenotypes, including concurrent mutations in other SCID genes, modifier gene(s), and mutations, that lead to sparing or disrupting developments of other lineages of lymphocytes. In addition, one patient with mutations in *RAG1* was classified as having B+ SCID with 267.4 CD19^+^ B cell/μL (Table 3). He likely had either Omenn syndrome or maternal engraftment due to his T+B+NK+ immunophenotype as well as his clinical presentation of severe eczema and eosinophilia (Table 3; Table SE4 in Supplementary Material). The T+B+NK+ immunophenotype of this patient may be explained by his missense mutation (c.2095C>T; p.R699W). This hypomorphic mutation results in a mutant RAG1 enzyme with 19.3% residual recombinase activity (53), thus allowing the generation of B cells in this patient. These cases in our study as well as previous reports demonstrated the imperfect correlation of genotype–immunophenotype in SCID patients.

We reported two patients (P091 and P092) with *RFXANK* mutations, with one (P092) reported previously in Chinese literature (54). These patients were the only two with confirmed *RFXANK* mutations reported in Asia. *RFXANK* mutation causes bare lymphocyte syndrome type 2B (55), commonly observed in North Africa (56) and sometimes in other places such as France and Spain (57). MHC class II deficiency accounted for 32% of all forms of SCID and their variants in North Africa (58–62), while it accounted for 1.4% of all SCID in Asia. Such discrepancy could be explained by the higher consanguinity rate of 50% in North Africa (63) compared to that of less than 10% in Asia (45), as well as presence of founder mutations in North Africa (56, 64). These patients present with features of typical SCID and frequently with sclerosing cholangitis (56, 65). However, they have normal TREC level and cannot be identified by newborn screening (5, 66).

The care of patients with SCID in Asia is still at an early phase of development, as reflected by delay in diagnosis and suboptimal management with no easy access to HSCT (12, 14). Eighty-one of the 83 patients were the first member of their respective families to be diagnosed genetically with SCID, thus explaining the relative lack of family history of SCID in our study. For genetic counseling, we offered testing for family members of SCID patients including prenatal and newborn screening on siblings of six index patients as well as carrier screening for parents, siblings, and maternal aunts of 56 index patients (Table SE6 in Supplementary Material). Unfortunately, there is still a relative lack of clinical genetic service in Asia.

The median ALC in all studies including ours was below 3 × 10^9^/L, and reaffirming lymphopenia is a feature commonly seen in SCID patients (Table 7). However, clinicians failed to act on this critical clue as lymphopenia did not affect the AD or time to diagnosis. Since 1994, many reports have emphasized the importance of low ALC in alerting clinicians regarding SCID (67–70), but sadly clinicians to this date still failed to appreciate the value of low ALC for the diagnosis of SCID.

In this study, we identified that FH, candidiasis, and ALC below 3 × 10^9^/L were overlooked clinical features prompting the diagnosis of SCID. In addition, BCG infections were useful clinical features as they were the second most common opportunistic infections in our SCID patients. Ninety-four percent of patients in our study had at least one of the following four features: FH, candidiasis, BCG infections, and ALC below 3 × 10^9^/L. Therefore, we suggest a simple guideline mandating that clinicians should order lymphocyte subset analysis for infants with any one of the following four features: FH, candidiasis, BCG infections, and ALC below 3 × 10^9^/L.

Failure to diagnose SCID in time will lead to delay in HSCT, leading to economic losses in addition to poor outcome. Study has shown that the mean total hospital charges in patients who had HSCT after 3.5 months were four times greater than those before 3.5 months (71). Since all the clinical features we analyzed failed to help clinicians in making earlier SCID diagnosis, newborn screening is the only solution for making early enough diagnosis of SCID for timely HSCT in Asia.

Our retrospective case-series relied on reports made by referring doctors instead of analyzing original charts and results; therefore, underreport of clinical features was possible. In addition, our handling of missing data tends to underestimate the strength of association of clinical features with AD and time to diagnosis.

In conclusion, clinicians failed to recognize typical clinical features of SCID to shorten the time to diagnosis. There is an urgent unmet need to educate clinicians in Asia on SCID. Ultimately, the only solution for early diagnosis of and timely HSCT for patients with SCID is newborn screening.

## Ethics Statement

Genetic and functional studies on PID, data archival in the APIN database, and DNA storage were approved by the Clinical Research Ethics Review Board of the University of Hong Kong and Queen Mary Hospital (Ref. no. UW 08-301).

## Author Contributions

ADL, PL, and YL designed the study; ADL and YL wrote the manuscript with extensive appraisal from PL, K-WC, and HM; YL, K-WC, WW, and ADL analyzed the data; K-WC, WY, and WT performed the genetic and immunological studies; PL, HM, YL, XC, T-XC, JH, NK, DS, YT, YX, LJ, WL, OJ, TD, AG, SS, AR, AHL, ACL, LS, TN, TC, YC, ZL, TML, NL, BL, QL, DR, M-RB, M-KT, MA, XW, CX, HY, H-HY, TLL, FY, PC, and MH contributed clinical data and took care of patients in this study. PL and YL established the Asian Primary Immunodeficiency Network (APIN).

## Conflict of Interest Statement

The authors declare that the research was conducted in the absence of any commercial or financial relationships that could be construed as a potential conflict of interest.

## Supplementary Material

Figure S1 Distribution of age at diagnosis and time to diagnosis of patients with genetic diagnosis (*n* = 83). Distribution of age at diagnosis of patients with chronic diarrhea **(A)**, severe infections **(B)**, and lymphopenia **(C)** and distribution of time to diagnosis of patients with chronic diarrhea **(D)** and lymphopenia **(E)**.

## Abbreviations

*ADA*, adenosine deaminase gene; ALC, absolute lymphocyte count; AD, age at diagnosis; AP, age at presentation; APIN, Asian Primary Immunodeficiency Network; BCG, bacillus Calmette–Guérin; CMV, cytomegalovirus; *DCLRE1C*, DNA cross-link repair enzyme 1C (Artemis); FH, family history of early infant death; FN3, fibronectin type-III; FTT, failure to thrive; HSCT, hematopoietic stem cell transplant; ICU, intensive care unit; *IL2RG*, IL-2 receptor gamma chain; *IL7R*, IL-7 receptor alpha chain; *JAK 3*, janus kinase 3; PCP, *Pneumocystis jiroveci*; PID, primary immunodeficiency; *RAG1*, recombinase activating gene 1; *RAG2*, recombinase activating gene 2; *RFXANK*, regulatory factor X associated ankyrin containing protein; SCID, severe combined immunodeficiency; TREC, T-cell receptor excision circle.
