# Supplementary material for: Family History of Early Infant Death Correlates with Earlier Age at Diagnosis But Not Shorter Time to Diagnosis for Severe Combined Immunodeficiency
Source: Front Immunol. 2017 Jul 12;8:808. doi: 10.3389/fimmu.2017.00808 (PMC5506088; doi:10.3389/fimmu.2017.00808)
Supplement: Supplementary file 1 [file table_1.docx]

***Supplementary Material***

**Family history of early infant death correlates with earlier age at diagnosis but not shorter time to diagnosis for severe combined immunodeficiency.**

**Anderson Dik Wai Luk^1^, Pamela P. Lee^1^, Huawei Mao^1,2^, Koon-Wing Chan^1^, Xiang Yuan Chen^3^, Tong-Xin Chen^4^, Jian Xin He^5^, Nadia Kechout^6^, Deepti Suri^7^, Yin Bo Tao^3^, Yong Bin Xu^8^, Li Ping Jiang^9^, Woei Kang Liew^10^, Orathai Jirapongsananuruk^11^, Tassalapa Daengsuwan^12^, Anju Gupta^7^, Surjit Singh^7^, Amit Rawat^7^, Amir Hamzah Abdul Latiff^13^, Anselm Chi Wai Lee^14^, Lynette P Shek^15^, Thi Van Anh Nguyen^16^, Tek Jee Chin^17^, Yin Hsiu Chien^18^, Zarina Abdul Latiff^19^, Thi Minh Huong Le^16^, Nguyen Ngoc Quynh Le^16^, Bee Wah Lee^15^, Qiang Li^20^, Dinesh Raj^21^, Mohamed-Ridha Barbouche^22^, Meow-Keong Thong^23^, Maria Carmen D. Ang^24^, Xiao Chuan Wang^25^, Chen Guang Xu^26^, Hai Guo Yu^27^, Hsin-Hui Yu^18^, Tsz Leung Lee^1^, Felix Yat Sun Yau^28^, Wilfred Hing-sang Wong^1^, Wenwei Tu^1,2^, Wangling Yang^1,2^, Patrick Chun Yin Chong^1^, Marco Hok Kung Ho^1^, Yu Lung Lau^1,2*^**

***Correspondence:** Yu Lung Lau, MD (Honors), Department of Paediatrics & Adolescent Medicine, Li Ka Shing Faculty of Medicine, the University of Hong Kong, Pokfulam Road, Hong Kong Special Administrative Region, PR China: [lauylung@hku.hk](mailto:lauylung@hku.hk)

**Supplementary table E1. Genetic mutations of SCID patients excluded in present study (n= 11)**

No. Gene Intron(I)/Exon(E) Nucleotide change Predicted change

P009 *IL2RG*  E3 c.304T>C C102R

P010 *IL2RG*  E3 c.306C>A C102X

P029 *IL2RG*  E5 c.618T>A H206Q

P035 *IL2RG* E5 c.676C>T R226C

P043 *IL2RG* E6 c.800T>A V267D

P054 *IL2RG*  I6/E7 Junction g.IVS6-2A>T Predicted aberrant splicing

P061 *IL2RG*  E8 c.979G>A E327K

P063b^1^ *IL2RG*  E8 c.982C>T R328X

P067^2^ *DCLRE1C* E1-E4 Gross Deletion Gross Deletion

P082^2^ *RAG1* E2 c.2073_2074insG M691fsX693

P090^2^  *RAG2*  E2 c.1219G>T E407X

^1^P063b and P063a (table I) were from the same kindred. ^2^ Homozygous mutations.
