# Supplementary material for: Family History of Early Infant Death Correlates with Earlier Age at Diagnosis But Not Shorter Time to Diagnosis for Severe Combined Immunodeficiency
Source: Front Immunol. 2017 Jul 12;8:808. doi: 10.3389/fimmu.2017.00808 (PMC5506088; doi:10.3389/fimmu.2017.00808)
Supplement: Supplementary file 2 [file table_2.docx]

***Supplementary Material***

**Family history of early infant death correlates with earlier age at diagnosis but not shorter time to diagnosis for severe combined immunodeficiency.**

**Anderson Dik Wai Luk^1^, Pamela P. Lee^1^, Huawei Mao^1,2^, Koon-Wing Chan^1^, Xiang Yuan Chen^3^, Tong-Xin Chen^4^, Jian Xin He^5^, Nadia Kechout^6^, Deepti Suri^7^, Yin Bo Tao^3^, Yong Bin Xu^8^, Li Ping Jiang^9^, Woei Kang Liew^10^, Orathai Jirapongsananuruk^11^, Tassalapa Daengsuwan^12^, Anju Gupta^7^, Surjit Singh^7^, Amit Rawat^7^, Amir Hamzah Abdul Latiff^13^, Anselm Chi Wai Lee^14^, Lynette P Shek^15^, Thi Van Anh Nguyen^16^, Tek Jee Chin^17^, Yin Hsiu Chien^18^, Zarina Abdul Latiff^19^, Thi Minh Huong Le^16^, Nguyen Ngoc Quynh Le^16^, Bee Wah Lee^15^, Qiang Li^20^, Dinesh Raj^21^, Mohamed-Ridha Barbouche^22^, Meow-Keong Thong^23^, Maria Carmen D. Ang^24^, Xiao Chuan Wang^25^, Chen Guang Xu^26^, Hai Guo Yu^27^, Hsin-Hui Yu^18^, Tsz Leung Lee^1^, Felix Yat Sun Yau^28^, Wilfred Hing-sang Wong^1^, Wenwei Tu^1,2^, Wangling Yang^1,2^, Patrick Chun Yin Chong^1^, Marco Hok Kung Ho^1^, Yu Lung Lau^1,2*^**

***Correspondence:** Yu Lung Lau, MD (Honors), Department of Paediatrics & Adolescent Medicine, Li Ka Shing Faculty of Medicine, the University of Hong Kong, Pokfulam Road, Hong Kong Special Administrative Region, PR China: [lauylung@hku.hk](mailto:lauylung@hku.hk)

**Supplementary table E2. Chi-square analysis on clinical features between X-linked and autosomal recessive SCID patients (n= 83).**

Positive features X-linked SCID (n= 57) Autosomal recessive SCID (n= 26) p-value

FTT 8 5 0.546

Chronic diarrhea 29 13 0.941

Recurrent infections 31 19 0.107

FH 22 7 0.301

CMV infection 6 2 0.685

BCG infection 14 5 0.592

Persistent candidiasis 17 10 0.436

Severe infections 26 13 0.710

Opportunistic infections 31 19 0.107

Pneumonia 38 15 0.430

Sepsis 10 8 0.175

Hepatosplenomegaly 8 4 0.871

Lymphopenia^1^ 43 19 0.694

FTT, failure to thrive; FH, family history of early infant death. ^1^ Defined as ALC below 3 x 10^9^/L.
