# Supplementary material for: Family History of Early Infant Death Correlates with Earlier Age at Diagnosis But Not Shorter Time to Diagnosis for Severe Combined Immunodeficiency
Source: Front Immunol. 2017 Jul 12;8:808. doi: 10.3389/fimmu.2017.00808 (PMC5506088; doi:10.3389/fimmu.2017.00808)
Supplement: Supplementary file 3 [file table_3.docx]

***Supplementary Material***

**Family history of early infant death correlates with earlier age at diagnosis but not shorter time to diagnosis for severe combined immunodeficiency.**

**Anderson Dik Wai Luk^1^, Pamela P. Lee^1^, Huawei Mao^1,2^, Koon-Wing Chan^1^, Xiang Yuan Chen^3^, Tong-Xin Chen^4^, Jian Xin He^5^, Nadia Kechout^6^, Deepti Suri^7^, Yin Bo Tao^3^, Yong Bin Xu^8^, Li Ping Jiang^9^, Woei Kang Liew^10^, Orathai Jirapongsananuruk^11^, Tassalapa Daengsuwan^12^, Anju Gupta^7^, Surjit Singh^7^, Amit Rawat^7^, Amir Hamzah Abdul Latiff^13^, Anselm Chi Wai Lee^14^, Lynette P Shek^15^, Thi Van Anh Nguyen^16^, Tek Jee Chin^17^, Yin Hsiu Chien^18^, Zarina Abdul Latiff^19^, Thi Minh Huong Le^16^, Nguyen Ngoc Quynh Le^16^, Bee Wah Lee^15^, Qiang Li^20^, Dinesh Raj^21^, Mohamed-Ridha Barbouche^22^, Meow-Keong Thong^23^, Maria Carmen D. Ang^24^, Xiao Chuan Wang^25^, Chen Guang Xu^26^, Hai Guo Yu^27^, Hsin-Hui Yu^18^, Tsz Leung Lee^1^, Felix Yat Sun Yau^28^, Wilfred Hing-sang Wong^1^, Wenwei Tu^1,2^, Wangling Yang^1,2^, Patrick Chun Yin Chong^1^, Marco Hok Kung Ho^1^, Yu Lung Lau^1,2*^**

***Correspondence:** Yu Lung Lau, MD (Honors), Department of Paediatrics & Adolescent Medicine, Li Ka Shing Faculty of Medicine, the University of Hong Kong, Pokfulam Road, Hong Kong Special Administrative Region, PR China: [lauylung@hku.hk](mailto:lauylung@hku.hk)

**Supplementary table E3. Analysis of unreported genetic mutations in present study.**

Mutation AA change Structure Population Other reported mutation Cosegregation with diseases Prediction of missense mutations effect on protein function

involved frequency on the same AA residue

PANTHER PHD-SNP SIFT SNAP Meta-SNP PolyPhen 2

**(IL2RG)**

127delA T43fsX70 0 Yes

202G>T E68X 0 E68K/E68G Yes

306C>A C102X 0 C102Y/C102R

340G>T G114C 0 G114D Yes + + + + + +

362delA E121fsX146 0 Yes

365T>C I122T 0 Yes - - + + - +

383T>C F128S 0 + + + + + +

386T>A V129D 0 Yes + - + + + +

406-415del R136fsX143 0 Yes

IVS3-2A>T PAS 0 Yes

618T>A H206Q FN3 0 Yes - - - + - -

658­­­_659del T220fsX227 FN3 0

741delG G247fsX272 FN3 0

741_742insG E248fsX302 FN3, Box1 0

800T>A V267D TM 0 V267L - + + + + +

811G>T G271X TM 0 G271E Yes

IVS6-2A>T PAS Box1 0 Yes

IVS6+3G>T PAS Box1 0 Yes

IVS6-2A>C PAS Box1 0

929G>A W310X 0

979G>A E327K 0 Yes + + + + + +

979-980delinsTT E327L 0 + + + + + +

982C>T R328X 0 Yes

**(JAK3)**

1763A>C H588P PK1 0 Yes + + + + + +

**(RAG1)**

2561G>A G854D 0 Yes - + + + + +

2073_2074insG M692fsX693 0 Yes

2324T>A L775Q 0 L775R - + + + + +

**(RAG2)**

475C>T R159C 0 R159C^1^ Yes + + + + + +

**(RFXANK)**

IVS3+1delG PAS 0 Yes

AA, amino acid; PAS, predicted aberrant splicing; FN3, fibronectin type-III domain; Box1, Box-1 motif; TM, transmembrane region; PK1, protein kinase 1 domain. ^1^ 476G>A causing R159C was previously reported [17]. +, Predicted disease-causing variants; -, predicted neutral variants.
