# Supplementary material for: Family History of Early Infant Death Correlates with Earlier Age at Diagnosis But Not Shorter Time to Diagnosis for Severe Combined Immunodeficiency
Source: Front Immunol. 2017 Jul 12;8:808. doi: 10.3389/fimmu.2017.00808 (PMC5506088; doi:10.3389/fimmu.2017.00808)
Supplement: Supplementary file 4 [file table_4.docx]

***Supplementary Material***

**Family history of early infant death correlates with earlier age at diagnosis but not shorter time to diagnosis for severe combined immunodeficiency.**

**Anderson Dik Wai Luk^1^, Pamela P. Lee^1^, Huawei Mao^1,2^, Koon-Wing Chan^1^, Xiang Yuan Chen^3^, Tong-Xin Chen^4^, Jian Xin He^5^, Nadia Kechout^6^, Deepti Suri^7^, Yin Bo Tao^3^, Yong Bin Xu^8^, Li Ping Jiang^9^, Woei Kang Liew^10^, Orathai Jirapongsananuruk^11^, Tassalapa Daengsuwan^12^, Anju Gupta^7^, Surjit Singh^7^, Amit Rawat^7^, Amir Hamzah Abdul Latiff^13^, Anselm Chi Wai Lee^14^, Lynette P Shek^15^, Thi Van Anh Nguyen^16^, Tek Jee Chin^17^, Yin Hsiu Chien^18^, Zarina Abdul Latiff^19^, Thi Minh Huong Le^16^, Nguyen Ngoc Quynh Le^16^, Bee Wah Lee^15^, Qiang Li^20^, Dinesh Raj^21^, Mohamed-Ridha Barbouche^22^, Meow-Keong Thong^23^, Maria Carmen D. Ang^24^, Xiao Chuan Wang^25^, Chen Guang Xu^26^, Hai Guo Yu^27^, Hsin-Hui Yu^18^, Tsz Leung Lee^1^, Felix Yat Sun Yau^28^, Wilfred Hing-sang Wong^1^, Wenwei Tu^1,2^, Wangling Yang^1,2^, Patrick Chun Yin Chong^1^, Marco Hok Kung Ho^1^, Yu Lung Lau^1,2*^**

***Correspondence:** Yu Lung Lau, MD (Honors), Department of Paediatrics & Adolescent Medicine, Li Ka Shing Faculty of Medicine, the University of Hong Kong, Pokfulam Road, Hong Kong Special Administrative Region, PR China: [lauylung@hku.hk](mailto:lauylung@hku.hk)

**Supplementary table E4. Characteristics of patients with genotype-immunophenotype miscorrelation.**

Patient Clinical presentation ALC(x10^9^/L) FH CD19+cell/uL(%) Mutation(Gene) Other SCID gene tested negative

P001 Lymphopenia 0.14 No 70(50) c.3G>T; p.M1I *(IL2RG)* Not done

P002 Lymphopenia, pneumonia, CMV infection 0.67 No 13.4(2) c.127delA; p.T43fsX70 *(IL2RG)* Not done

P003 Chronic diarrhea, FTT, disseminated BCG 3.6 Yes^2^ 0(0) c.202G>T; p.E68X *(IL2RG)* Not done

P005 Recurrent infections 0.18 No 3.6(2) c.202G>A; p.E68K *(IL2RG)* *DCLRE1C, RAG1, RAG2*

P078^1^ Severe eczema, RTI, recurrent salmonella GE, 7.64 No 267.4(3.5) c.1178delG; p.G393fsX402 *(RAG1)* Not done

recurrent herpes zoster, eosinophilia c.2095C>T; p.R699W *(RAG1)*

B- SCID was defined as having <134 CD19+ cells/uL. c. indicates nucleotide changes; p. indicates predicted changes in protein. ALC, absolute lymphocyte count; FH, family history of early infant death; CMV, cytomegalovirus; FTT, failure to thrive; RTI, respiratory tract infection; GE, gastroenteritis. Lymphopenia was defined as ALC below 3 x 10^9^/L. ^1^P078 had double heterozygous mutation of *RAG1*. ^2^P003 had family history of early infant death of 5 maternal uncles.
