# Supplementary material for: Family History of Early Infant Death Correlates with Earlier Age at Diagnosis But Not Shorter Time to Diagnosis for Severe Combined Immunodeficiency
Source: Front Immunol. 2017 Jul 12;8:808. doi: 10.3389/fimmu.2017.00808 (PMC5506088; doi:10.3389/fimmu.2017.00808)
Supplement: Supplementary file 5 [file table_5.docx]

***Supplementary Material***

**Family history of early infant death correlates with earlier age at diagnosis but not shorter time to diagnosis for severe combined immunodeficiency.**

**Anderson Dik Wai Luk^1^, Pamela P. Lee^1^, Huawei Mao^1,2^, Koon-Wing Chan^1^, Xiang Yuan Chen^3^, Tong-Xin Chen^4^, Jian Xin He^5^, Nadia Kechout^6^, Deepti Suri^7^, Yin Bo Tao^3^, Yong Bin Xu^8^, Li Ping Jiang^9^, Woei Kang Liew^10^, Orathai Jirapongsananuruk^11^, Tassalapa Daengsuwan^12^, Anju Gupta^7^, Surjit Singh^7^, Amit Rawat^7^, Amir Hamzah Abdul Latiff^13^, Anselm Chi Wai Lee^14^, Lynette P Shek^15^, Thi Van Anh Nguyen^16^, Tek Jee Chin^17^, Yin Hsiu Chien^18^, Zarina Abdul Latiff^19^, Thi Minh Huong Le^16^, Nguyen Ngoc Quynh Le^16^, Bee Wah Lee^15^, Qiang Li^20^, Dinesh Raj^21^, Mohamed-Ridha Barbouche^22^, Meow-Keong Thong^23^, Maria Carmen D. Ang^24^, Xiao Chuan Wang^25^, Chen Guang Xu^26^, Hai Guo Yu^27^, Hsin-Hui Yu^18^, Tsz Leung Lee^1^, Felix Yat Sun Yau^28^, Wilfred Hing-sang Wong^1^, Wenwei Tu^1,2^, Wangling Yang^1,2^, Patrick Chun Yin Chong^1^, Marco Hok Kung Ho^1^, Yu Lung Lau^1,2*^**

***Correspondence:** Yu Lung Lau, MD (Honors), Department of Paediatrics & Adolescent Medicine, Li Ka Shing Faculty of Medicine, the University of Hong Kong, Pokfulam Road, Hong Kong Special Administrative Region, PR China: [lauylung@hku.hk](mailto:lauylung@hku.hk)

**Supplementary table E5. BCG vaccination policies of countries and regions in present study, Brazil and Iran.**

Country Immunization schedule^1^ BCG strain used^2^ Virulence^3^ Population coverage(%)^1^

Algeria At birth or within 4 weeks N/A N/A 99%

China At birth China^4^ Intermediate 99%

Hong Kong At birth Russian Intermediate 99%

India At birth Danish Intermediate 99%

Indonesia 1 month Pasteur 1173 P2 High 92.2%

Korea At birth Danish Intermediate 85%

Malaysia At birth Tokyo Low 99%

Philippine At birth N/A N/A 80%

Pakistan At birth Tokyo Low 95%

Singapore At birth Danish Intermediate 97%

Taiwan At birth Tokyo Low 98.4%

Thailand At birth Tokyo Low 99%

Tunisia At birth Pasteur 1173 P2 High >95%

Vietnam At birth Pasteur 1173 P2 High 93.7%

Brazil At birth Moreau Intermediate 99%

Iran At birth Pasteur 1173 P2 High 99%

^1^ From BCG World Atlas database [34] and UNICEF [35]. ^2^ From BCG World Atlas database [34] and published data [30, 36, 37, 38]. ^3^ Virulence in SCID mice [39]. ^4^ Derived from Danish strain [37].
