# Supplementary material for: Family History of Early Infant Death Correlates with Earlier Age at Diagnosis But Not Shorter Time to Diagnosis for Severe Combined Immunodeficiency
Source: Front Immunol. 2017 Jul 12;8:808. doi: 10.3389/fimmu.2017.00808 (PMC5506088; doi:10.3389/fimmu.2017.00808)
Supplement: Supplementary file 6 [file table_6.docx]

***Supplementary Material***

**Family history of early infant death correlates with earlier age at diagnosis but not shorter time to diagnosis for severe combined immunodeficiency.**

**Anderson Dik Wai Luk^1^, Pamela P. Lee^1^, Huawei Mao^1,2^, Koon-Wing Chan^1^, Xiang Yuan Chen^3^, Tong-Xin Chen^4^, Jian Xin He^5^, Nadia Kechout^6^, Deepti Suri^7^, Yin Bo Tao^3^, Yong Bin Xu^8^, Li Ping Jiang^9^, Woei Kang Liew^10^, Orathai Jirapongsananuruk^11^, Tassalapa Daengsuwan^12^, Anju Gupta^7^, Surjit Singh^7^, Amit Rawat^7^, Amir Hamzah Abdul Latiff^13^, Anselm Chi Wai Lee^14^, Lynette P Shek^15^, Thi Van Anh Nguyen^16^, Tek Jee Chin^17^, Yin Hsiu Chien^18^, Zarina Abdul Latiff^19^, Thi Minh Huong Le^16^, Nguyen Ngoc Quynh Le^16^, Bee Wah Lee^15^, Qiang Li^20^, Dinesh Raj^21^, Mohamed-Ridha Barbouche^22^, Meow-Keong Thong^23^, Maria Carmen D. Ang^24^, Xiao Chuan Wang^25^, Chen Guang Xu^26^, Hai Guo Yu^27^, Hsin-Hui Yu^18^, Tsz Leung Lee^1^, Felix Yat Sun Yau^28^, Wilfred Hing-sang Wong^1^, Wenwei Tu^1,2^, Wangling Yang^1,2^, Patrick Chun Yin Chong^1^, Marco Hok Kung Ho^1^, Yu Lung Lau^1,2*^**

***Correspondence:** Yu Lung Lau, MD (Honors), Department of Paediatrics & Adolescent Medicine, Li Ka Shing Faculty of Medicine, the University of Hong Kong, Pokfulam Road, Hong Kong Special Administrative Region, PR China: [lauylung@hku.hk](mailto:lauylung@hku.hk)

**Supplementary table E6. Genetic testing for family members of SCID patients (n= 83).**

**Genetic testing^1^ Frequency (%)**

**Carrier testing**

Parents

Both 46 (55.4)

Mother only 9 (10.8)

Father only 1 (1.2)

Not screened 27 (32.5)

Siblings 5 (6.0)

Maternal aunt 2 (2.4)

**Prenatal or newborn screening**

Prenatal only 3 (3.6)

Newborn only 1 (1.2)

Prenatal and newborn 2 (2.4)

^1^ We offered genetic testing on *IL2RG, IL7R, JAK3, ADA, DCLRE1C, RAG1, RAG2* and *RFXANK* for family members of SCID patients based on the genetic mutations found on those patients.
