# Supplementary material for: Family History of Early Infant Death Correlates with Earlier Age at Diagnosis But Not Shorter Time to Diagnosis for Severe Combined Immunodeficiency
Source: Front Immunol. 2017 Jul 12;8:808. doi: 10.3389/fimmu.2017.00808 (PMC5506088; doi:10.3389/fimmu.2017.00808)
Supplement: Supplementary file 7 [file data_sheet_1.docx]

***Supplementary Material***

**Family history of early infant death correlates with earlier age at diagnosis but not shorter time to diagnosis for severe combined immunodeficiency.**

**Anderson Dik Wai Luk^1^, Pamela P. Lee^1^, Huawei Mao^1,2^, Koon-Wing Chan^1^, Xiang Yuan Chen^3^, Tong-Xin Chen^4^, Jian Xin He^5^, Nadia Kechout^6^, Deepti Suri^7^, Yin Bo Tao^3^, Yong Bin Xu^8^, Li Ping Jiang^9^, Woei Kang Liew^10^, Orathai Jirapongsananuruk^11^, Tassalapa Daengsuwan^12^, Anju Gupta^7^, Surjit Singh^7^, Amit Rawat^7^, Amir Hamzah Abdul Latiff^13^, Anselm Chi Wai Lee^14^, Lynette P Shek^15^, Thi Van Anh Nguyen^16^, Tek Jee Chin^17^, Yin Hsiu Chien^18^, Zarina Abdul Latiff^19^, Thi Minh Huong Le^16^, Nguyen Ngoc Quynh Le^16^, Bee Wah Lee^15^, Qiang Li^20^, Dinesh Raj^21^, Mohamed-Ridha Barbouche^22^, Meow-Keong Thong^23^, Maria Carmen D. Ang^24^, Xiao Chuan Wang^25^, Chen Guang Xu^26^, Hai Guo Yu^27^, Hsin-Hui Yu^18^, Tsz Leung Lee^1^, Felix Yat Sun Yau^28^, Wilfred Hing-sang Wong^1^, Wenwei Tu^1,2^, Wangling Yang^1,2^, Patrick Chun Yin Chong^1^, Marco Hok Kung Ho^1^, Yu Lung Lau^1,2*^**

***Correspondence:** Yu Lung Lau, MD (Honors), Department of Paediatrics & Adolescent Medicine, Li Ka Shing Faculty of Medicine, the University of Hong Kong, Pokfulam Road, Hong Kong Special Administrative Region, PR China: [lauylung@hku.hk](mailto:lauylung@hku.hk)

**Supplementary E1. Genomic PCR Sequencing for SCID genes.**

Genomic PCR, DNA sequencing and homology analysis were explained in our previous study [13], here we listed the primers used.

IL2RG

Exon Sense Primer 5’🡪3’ Anti-sense Primer 5’🡪3’

1 GGGTGACCAAGTCAAGGAAGA CCACATGATTGTAATGGCCAGT

2-3 GTGCTTGGCCTCCTCCTTCT TCTTTTCTGCCCATGTACCCTT

3-4 TGGGGTAAGTCATAAGTCGGTT TTAGGTCCTTCTATCTGTCTGG

5 TGGCTTGAGTAGTCAAGAGATG GGAGAGATGGGGCACCAAGT

6-7 AGGTAATTGTTCCTGCTCACA CACATCCTGACAGTTAGTGCA

8 GAGCCTGTGTATCCCTTCTG CATCGGTTCAGGAACAATCGGA

ADA

Exon Sense Primer 5’🡪3’ Anti-sense Primer 5’🡪3’

1 TTCCAGGAAATGCGCGATCCA GTTCGTTCCCAGGGTTTGGAC

2 AACATTAAGCTCTGAAAGGTCC ATTCCCACAGGGAGACAGGAA

3 TGCTGTGGTCCTGCAAGGAG CAGCTGTGGTTTCAGAGCA

4 ATCCAGTGGATGCCCAGAGCT TGAGGCCATGGACCAGACTG

5 CTCTGAGGCAGTGGGTCAGT TTCATGCCAGTGGGCTCAAGG

6 GTGGTTAGGACATAGCAGTTAG CCCTGGTTCTTGTGATTTCTCC

7-9 CAGGTTTCCCCATGCTGTTGA GATGCCCAATCCCTAAAGTTTC

10 CAGATCTGTTTGAGGCTGCTG CCTCTCTCCAAAGATTCCAGG

11 GCCCATGTTGAGTGAAGAGGA GAAACCAGGGAGTCATCACAC

12 CTTTCTGGTTCTGGTGTTCTA TGCTGCTGAGCAGGCTGACC

DCLRE1C

Exon Sense Primer 5’🡪3’ Anti-sense Primer 5’🡪3’

1 CAAGCAGGAAGCGGAACGAAG AGCCTTTGAGAGGCTGAAGAG

2 CATGGGTTTTCTCTTGAAGCTG GGTCTGGGATATTGGTACTGTC

3 CAGAAGTTTCTGTTCTCAACACAC CATGTCCGGCCAGAGACTCA

4 CGGAATTGTTTATATAGAGAGTCTG GAAAGATTGAGGGTATAGCTGAG

5 CTAGACCACATTTTCCTGATTGA ATTACAGACATGTGCCACTGCA

6 CACATGTATTTGCTTTATTGGGAG CACTAAAAATACAAAATTAGCTGGGC

7-8 TTAGCAAGGTGTGGTGGCACAA CATGGTGAAATGTCGTCTGTGC

9 ATCCCTGAGCCGAGTGTCCA GATCTGTGTCTATTTACTAGTGAC

10 GTCTGGAAACTTTTCTACTTTTGT GGGCTTAGGCTAGACTGTGC

11 AAATGAGAAGGGCCAGCTTTGC CAGAGATGCTTCTGAGAGTCAG

12 GAGAACCACAGCCATAAGAGAG GAGTATCAGTTCAGAAATGTCCCA

13 GTAATACTATCAATGATGTCTACC ACCATGTGTCAAGTCTGACCTG

14 TCCCTTTGAAGAAGAGTGGCCT TGGTTGCTCTAGGTTGAAACG

IL7R

Exon Sense Primer 5’🡪3’ Anti-sense Primer 5’🡪3’

1 GCCTAGGTGTGAATCTACTGCC TCAAACCCAGTGCCTGACTCA

2 CCCTTGGGCTTTTCTTCCTTG GAGTTTCAGGAGGCCTTTGGGA

3 CACTCACCCACCCACATACCTA TCAGAATGTTGTGGGGATTTGG

4 GCTGGAGAATGCGGACTGGA TATGCATCAGGGAAGATCCAGG

5 CTCCTTTACGTATCAGAGCTCC GCCCTCCAAGGGTGTCCTAT

6 GGCCTGGTCACCCAAGTCAA GGATAGATAGGGATACTGGGCAC

7 GAGGCCCAGAGAAAGCCAGTC GTTCCCTACTGCAGCTAGGGTC

8 CAATTCTGTGACATTCCCTGTCA TCTGTGCTGTGAGGGAGACTAGG

JAK3

Exon Sense Primer 5’🡪3’ Anti-sense Primer 5’🡪3’

1 CAAATCCAGCCAGGTTTCCTTC TAGCCTTTGCCCTGGCAGCA

2 CCTCCAGCACTCCTTTCCATG CACACCTCTCCGTCTGCACA

3 GGTGTGCAGGGTTGGCTTCT CCTCATCTGAGAAATGGGTAGTG

4-5 TCCCTCGCCCCCACCATAA CCCACGTTGCTCACTCCCAA

6 TGTGCCTGGTGCCCCAACTA CCCACTTCCCCAAGTCTTTCG

7-8 GGCAAAGGGATAGGGAGTGGA CCTGTCCAGGGCTCCTGGAA

9 GGTGTCACCTGGCAAGGATC CTTCAGGAACCAAATGCTGACT

10 CAGAGCCAGACTCCGTCTCCA TAACCCACCTTGACCTGCAGT

11-12 GTGCCCTGAAGTCTTCATCTC AAATTTCTCTGCATCCACGACC

13 GTGACCTGTGGCCAGGTGTT AATGAACACGGCTCCCATCCA

14 CAGAGATGAGAGTTTGAGAGAC CTCGAACCCTTACCAAACTCCT

15-16 CATGTCTGAGCAGTACCAAGTG TGTCAAAGTGGGGGTTCGGA

16-17 GGGCAAGGAAGTGGATCCCT CTGCAAACCACGCTCCTTCCA

18-19 CATAATGCACAGAGAGGGTCAA GTAACCCATGTGAATCTGGATC

20-21 CAAGGTCCCACTGTGAAAGG CTAAGGCTGGGGAGCAAAGCA

22-23 TGACCAGTTCCCCATTCCAAG CCTTTCTTCTCAGTACAGAGAC

24 CTGAATGGGAGTTGTGTCCTTTG CAGGGCTTAAGGGGTTGGGAA

RAG1

Exon Sense Primer 5’🡪3’ Anti-sense Primer 5’🡪3’

1 ACCAGGTTGAAAGGTTCTGAGA TGCTCATTCCATCCCCTCATCA

2A AACTCTATGATCAGCACCTAAC CGGGAAGTAAACCTCACATGG

2B CCAAGGTTTTCCGGATCGATG AAGGCTTTGACTTGCAGCTTG

2C TCACCACATCTCAAGTCACAAG TCTGAGAGCTGTGGGCAATAG

2D GGTGGTGAAGGAGTCTTGTG CTCTTTGGTCATGAGCTTCCTG

2E AATCCCAATGCTTCCAAAGAGG CCATACACAGCAGTAAAGGGT

RAG2

Exon Sense Primer 5’🡪3’ Anti-sense Primer 5’🡪3’

1 GCCTGATATAAAGTTGGTAGAG ACACTGCCAAAATGTGGCATCT

2A TGCTATTCACATGTGAAGGAATC GGCAGGCAGTCAGCTACACT

2B TCCTGAAGCCAGATATGGTCAT TGTTTGTGAATGTTGTCTGCTCT

2C GCAAGATATGGTTTGGAAGCAACA CATACACCTGAATCTGAAAGGCT

RFXANK

Exon Sense Primer 5’🡪3’ Anti-sense Primer 5’🡪3’

1 CTTTCTTCCCCTTGAGCTGCA AGCCAATTGGCTCCGGCTTC

2 GGGCCAGAGGCAGCACTTAC TATTCGCTATGGTTGTTGGGAG

3 CTGGTAATTAACCTGGACCTCA TCCATGTGAGGCCTGACATTC

4-6 AACAGATGGGCTTCTGTCCTG CAGCCGCATCTCAAAGACAAGG

7 AGATGCGGCTGCTGTGGGTA GAATGGGGCTGTCTCGTGTTC

8-9 ATGGGCCCAGTGATGAGCAG CTCAACACCTGTCAACACACTC

10 TCTGTAATGCAGGTCTCTGCAA GTGTGGTCATCTACCACCTGC
