# Supplementary figures and images for: Family History of Early Infant Death Correlates with Earlier Age at Diagnosis But Not Shorter Time to Diagnosis for Severe Combined Immunodeficiency
Source: Front Immunol. 2017 Jul 12;8:808. doi: 10.3389/fimmu.2017.00808 (PMC5506088; doi:10.3389/fimmu.2017.00808)

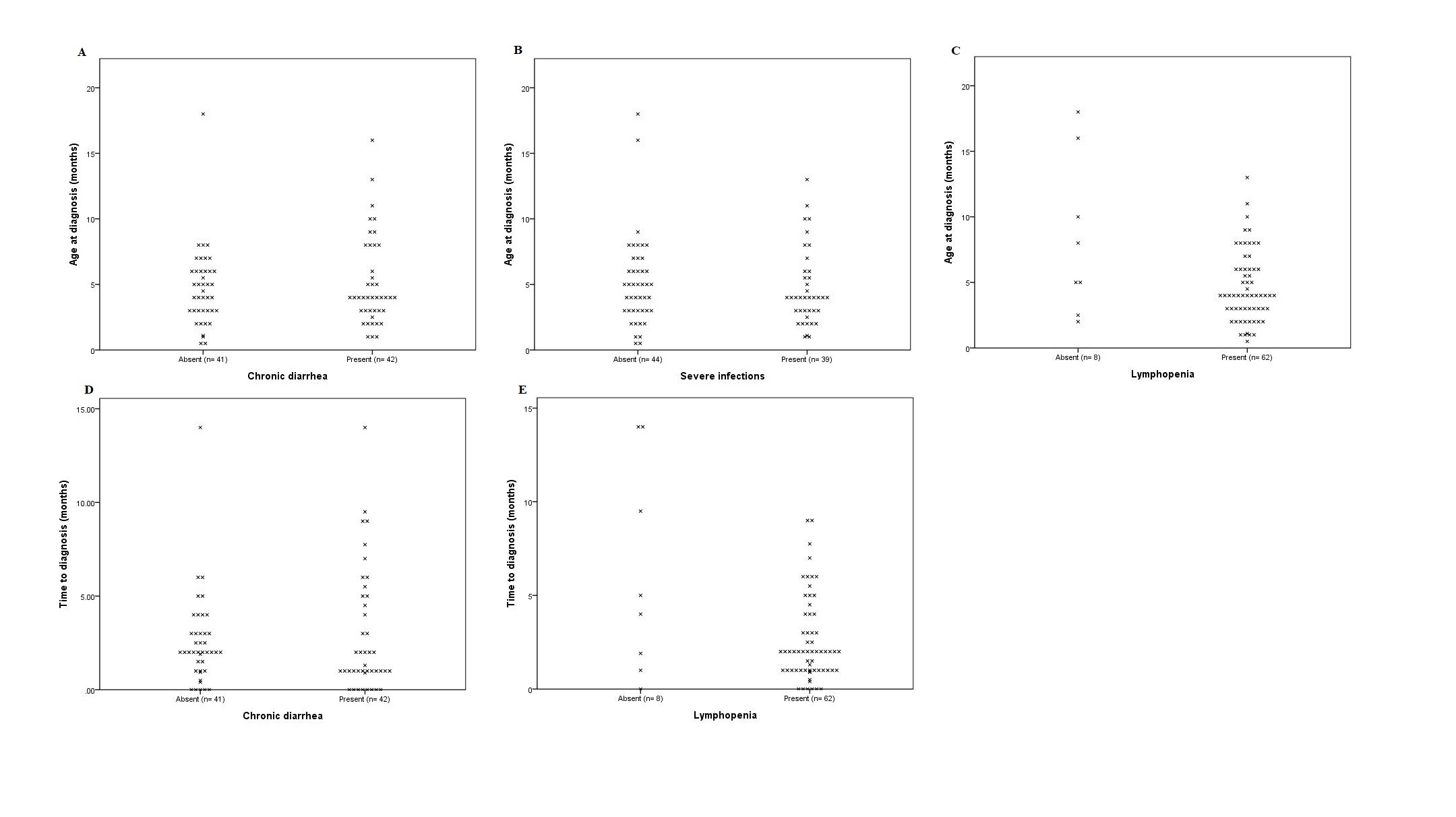

Supplement: Figure S1 — Distribution of age at diagnosis and time to diagnosis of patients with genetic diagnosis (n = 83). Distribution of age at diagnosis of patients with chronic diarrhea (A), severe infections (B), and lymphopenia (C) and distribution of time to diagnosis of patients with chronic diarrhea (D) and lymphopenia (E). [file image_1.jpeg]
